# An Update on Reported Variants in the Skeletal Muscle *α*-Actin (*ACTA1*) Gene

**DOI:** 10.1155/2024/6496088

**Published:** 2024-10-28

**Authors:** Joshua S. Clayton, Mridul Johari, Rhonda L. Taylor, Lein Dofash, Georgina Allan, Gavin Monahan, Peter J. Houweling, Gianina Ravenscroft, Nigel G. Laing

**Affiliations:** ^1^Harry Perkins Institute of Medical Research, QEII Medical Centre, Nedlands, Western Australia, Australia; ^2^Centre for Medical Research, University of Western Australia, QEII Medical Centre, Nedlands, Western Australia, Australia; ^3^Folkhälsan Research Center, Department of Medical and Clinical Genetics, Medicum, University of Helsinki, Helsinki, Finland; ^4^Murdoch Children's Research Institute, The Royal Children's Hospital, Melbourne, Victoria, Australia; ^5^Department of Pediatrics, University of Melbourne, The Royal Children's Hospital, Melbourne, Victoria, Australia

**Keywords:** ACTA1, actin, mutation update, nemaline myopathy, neuromuscular disease, rare disease, variants

## Abstract

The *ACTA1* gene encodes skeletal muscle alpha-actin, which forms the core of the sarcomeric thin filament in adult skeletal muscle. ACTA1 represents one of six highly conserved actin proteins that have all been associated with human disease. The first 15 pathogenic variants in *ACTA1* were reported in 1999, which expanded to 177 in 2009. Here, we update on the now 607 total variants reported in LOVD, HGMD, and ClinVar, which includes 343 reported pathogenic/likely pathogenic (P/LP) variants. We also provide suggested *ACTA1*-specific modifications to ACMG variant interpretation guidelines based on our analysis of known variants, gnomAD reports, and pathogenicity in other actin isoforms. Using these criteria, we report a total of 447 P/LP *ACTA1* variants. From a clinical perspective, the number of reported *ACTA1* disease phenotypes has grown from five to 20, albeit with some overlap. The vast majority (74%) of *ACTA1* variants cause nemaline myopathy (NEM), but there are increasing numbers that cause cardiomyopathy and novel phenotypes such as distal myopathy. We highlight challenges associated with identifying genotype–phenotype correlations for *ACTA1*. Finally, we summarize key animal models and review the current state of preclinical treatments for *ACTA1* disease. This update provides important resources and recommendations for the study and interpretation of *ACTA1* variants.

## 1. Introduction

Skeletal muscle alpha-actin (ACTA1) is a critical protein required for skeletal muscle structure and function. Pathogenic variants in *ACTA1* were first described in 1999 [1]. As of the last review in 2009, there were 177 reported pathogenic/likely pathogenic (P/LP) *ACTA1* variants [2]. Herein, we update on the now over 600 reported *ACTA1* variants, including 343 reported P/LP variants—an average of 11.9 new variants per year over the last 14 years. All aspects of *ACTA1* biology including gene structure, regulation of protein folding, posttranslational modification (PTM), and binding partners can inform the potential functional impacts of variants and therefore aid their classification.

*ACTA1* is 2.8 kb, located on chromosome 1q42.13, and encodes a 377 amino acid protein. Following posttranslational cleavage of the first two amino acid residues by ACTMAP protease [3], the protein folds into globular monomeric G-actin [4]. Each G-actin monomer contains binding sites that mediate head-to-tail interactions with two other actin monomers, which polymerize to form filamentous actin (F-actin). This process is controlled by ATP hydrolysis, ions, and multiple actin-binding proteins including cofilin and profilin [4].

In skeletal muscle, ACTA1 polymers interact with nebulin, troponins, and tropomyosins to form the core of the thin filament [5]. The functional unit of the thin filament (the sarcomere) consists of seven actin monomers, one troponin complex, and one tropomyosin molecule [6]. Following Ca^2+^ binding to tropomyosin, ACTA1 is bound by myosin in the thick filament, ultimately leading to muscle contraction [7]. In addition to myosin, actin is estimated to interact with > 100 other proteins [8], each of which can influence the assembly, conformation, and stability of actin. Associations with various cations (K^+^, Mg^2+^, and Ca^2+^) and nucleotides (ADP and ATP) also affect actin filament conformation [9, 10]. The competitive nature and complexity of these numerous interactions make it difficult to map discrete protein binding domains and identify clear disease mechanisms for many variants.

The phenotypic spectrum and severity of disease-causing variants in *ACTA1* are highly diverse, which presents numerous challenges for variant classification. In this mutation update, we summarize all *ACTA1* variants reported to date, with a particular focus on novel variants and phenotypes described since the previous mutation update [2]. We have standardized pathogenicity interpretation of all variants per ACMG guidelines using VarSome and provide further gene-specific comments and suggestions that may inform future classification of *ACTA1* variants.

## 2. Materials and Methods

### 2.1. ACTA1 Variant Curation

Variants were exported from LOVD3 (https://databases.lovd.nl/shared/genes/ACTA1), HGMD Pro (v2022.4), and ClinVar, all current as of 7 February 2023. A small number of additional variants were manually curated from the literature, as well as abstracts and/or personal communications. The final curation date for such additional variants was 6 August 2024, although only the original list of variants from LOVD, HGMD, and ClinVar was used for detailed analysis (see below methods). gnomAD variants were exported from both v2.1.1 and v3.1 datasets. All variants were processed using Ensembl Variant Effect Predictor (VEP) to standardize using the Human Genome Variation Society (HGVS) nomenclature. It is important to note that two numbering schemes have historically been used for ACTA1 mutations (amino acid changes). Classical numbering is based on the mature protein and therefore excludes the first two cleaved amino acids. This can cause confusion where variants are described solely by their amino acid change. In this update, tabulated variants are numbered using both systems since early publications use the classic mature protein numbering which can make variants difficult to find. In-text, variants are numbered according to the HGVS guidelines (https://varnomen.hgvs.org/). We use the preferred term “variant” here to describe genetic changes, though in select instances, we use the more traditional term “mutation” to specifically describe pathogenic changes that alter the resulting protein.

### 2.2. Variant Analysis and Classification

Compiled variants were analyzed in bulk using the VarSome Premium API [11] (https://varsome.com/) to unify pathogenicity calls between datasets and classify variants according to the ACMG guidelines [12]. As VarSome incorrectly classified some pathogenic variants as likely pathogenic or variants of uncertain significance (VUS) due to missing data, we uploaded data for 41 such variants to ClinVar to enable more complete and accurate variant interpretation by VarSome or manual classification in the future (submissions not approved at time of analysis). Variants were also analyzed using SpliceAI [13].

### 2.3. Minigene Assays

Minigene assays were used to assess the functional impact of select VUS that had previously been hypothesized by Laing et al. to impact splicing [2]. Assays were conducted as described previously [14, 15].

## 3. *ACTA1* Variants

### 3.1. Summary of Reported ACTA1 Variants

**Figure 1:**
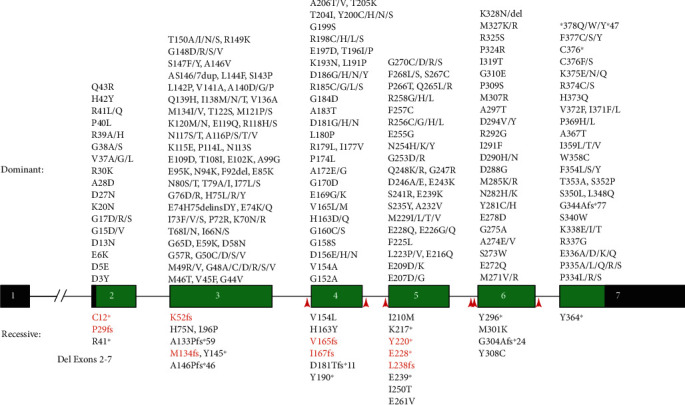
Pathogenic/likely pathogenic variants in the human *ACTA1* gene. Schematic of the human *ACTA1* gene showing the location of all known pathogenic dominant (above) and recessive (below) amino acid changes, as reported in HGMD, LOVD, and/or ClinVar. Coding exons (2–7) are in green, and UTRs are in black. Variants in orange are suspected recessive variants (ClinVar variants for which allele status is not stated) on the basis that they are frameshift or stop-gain changes not in either of the terminal exons. Red arrows indicate noncoding pathogenic variants that impact splicing.

We have collated a total of 607 *ACTA1* variants across HGMD, LOVD, and ClinVar, including 343 reported P/LP variants that affect 154/377 (40.8%) of ACTA1 residues. *ACTA1* variants are distributed evenly throughout the entire gene with no specific hotspots (Figure 1 and Table 1). Additional *ACTA1* variants identified since our original data collection can be found in Supporting Information 2 (Sheets 2.10 and 2.11) (current on 6 August 2024), which brings the total number of reported P/LP *ACTA1* variants to 350. These additional variants (in Supporting Information 2 (Sheets 2.10 and 2.11)) were not included in the analyses described herein. In addition to variants collated from other sources, we also report one novel de novo *ACTA1* variant, c.980T>G (p.Met327Arg), described in Supporting Information 1. The final curated and simplified list of 607 *ACTA1* variants can be found in Supporting Information 2 (Sheet 2.0). The detailed list (Supporting Information 2 (Sheet 2.1)) contains additional details and may be more useful for some readers.

To standardize pathogenicity calls and circumvent issues from conflicting reports of pathogenicity, we ran all 607 *ACTA1* variants through VarSome, which analyzes variants against ACMG guidelines. This resulted in 391 P/LP variants, 58 VUS, and 147 benign or likely benign (B/LB) variants. The remaining 11 variants were larger changes such as deletions that could not be classified by VarSome. Of the P/LP variants, 225 are new since the previous mutation update [2]. Given the large number of variants, we have presented these as Supporting Information (Supporting Information 2) which also includes smaller tables for assessment of variant pathogenicity, P/LP variants, VUS, B/LB variants, and phenotypes.

The majority of P/LP *ACTA1* variants are dominant (365/391, 93.3%), while the remainder are recessive (26/391, 6.6%) (Figure 1 and Supporting Information 4 (Sheet 4.2)). Although there are some reports of dominantly inherited *ACTA1* variants (*n* = 22), a much larger number arise de novo (*n* = 161 variants with confirmed reports). Therefore, of the dominant variants with confirmed reports, 88% are de novo. Most P/LP variants are missense (340/391, 87.0%), followed by frameshift (*n* = 18, 4.6%), nonsense (*n* = 13, 3.3%), splicing (*n* = 9, 2.3%), in-frame (*n* = 6, 1.5%), stop loss (*n* = 3, 0.8%), and start loss (*n* = 2, 0.5%). Reported stop codon variants (c.1134G>T, c.1133A>G, and c.1132T>C) all cause stop codon loss and inclusion of an additional 47 amino acids [16]. Generally, variants that cause a frameshift and/or premature termination are recessive, aside from variants that cause premature termination within the last 50 or so bases of Exon 6 or in Exon 7, which may escape nonsense-mediated decay and hence cause dominant disease [17]. There is one particularly interesting case (c.1031delG and p.Gly344Alafs∗77) of dominant disease inherited from an apparently unaffected father [18]. Incredibly, despite scrambling the last 34 residues and adding a further 44 additional amino acids, the patient is only mildly affected. In support of such actin being functional, mice expressing ACTA1 fused to EGFP demonstrate that additional sequence (albeit connected by a short linker) still enables actin to integrate into sarcomeres and form functional myofibres [19].

Almost all P/LP *ACTA1* variants are within the coding region (382/391, 97.7%). All pathogenic noncoding variants impact splicing (*n* = 9, 2.3%). Of these, six are known recessive variants that affect canonical splice positions (−2, −1, +1, and +2), plus another two (c.455-2A>T and c.455-2A>G) that are also likely recessive (status unconfirmed). There is only one reported dominant splice variant (c.617-5C>A), which is predicted to create a cryptic splice site and add one amino acid (Ala) translated from Intron 4 to the mature protein [2]. In our previous mutation update [2], we discussed that the c.210G>A (p.Lys70=) VUS reported by Graziano et al. [20] may affect splicing, but this could not be confirmed due to lack of patient muscle. We have since analyzed this variant using a minigene assay but found no evidence that this variant has any impact on splicing (Supporting Information 1). Together with ACMG interpretation, we classify this variant as likely benign.

**Figure 2:**
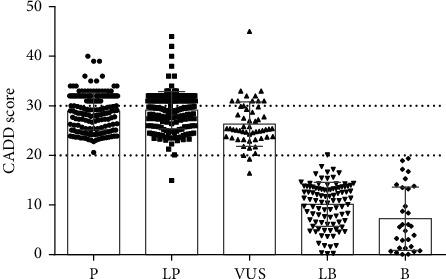
Pathogenicity calls for all *ACTA1* variants against the CADD score. CADD scores for the 596 *ACTA1* variants that could be analyzed/interpreted using VarSome. Error bars represent mean ± standard deviation. CADD scores of 20 (typical cut-off recommendation for pathogenic variants) and 30 (predicted 0.1% most deleterious substitutions in the human genome) are indicated by dotted lines.

All P/LP *ACTA1* missense variants have CADD scores > 20 (average 29.02), and known splice variants have SpliceAI scores ≥ 0.2 (average 0.88, *n* = 9). In contrast, all but one of the 147 B/LB variants have CADD scores < 20 (average 9.44), and only five B/LB variants have SpliceAI scores ≥ 0.2: c.809-9G>A (0.99), c.1040G>T (0.31), c.1065G>A (0.24), c.616+4C>T (0.21), and c.130-11C>G (0.2). Therefore, CADD and SpliceAI appear to have good predictive capability for distinguishing P/LP and B/LB variants in *ACTA1* (Figure 2), but as with all in silico tools, they should still be considered predictions which require empirical validation. It should also be noted that all but one B/LB variant is synonymous or intronic. The only exception is c.15C>G (p.Asp5Glu) which substitutes a highly similar residue that is naturally found in wild-type ACTC1 and other actin isoforms. On this basis, the substitution of almost any ACTA1 residue should generally be considered likely pathogenic.

### 3.2. VUS

There are 58 VUS in *ACTA1* based on VarSome classification (Supporting Information 1). However, we believe many of these 58 variants have likely been misclassified by VarSome due to missing phenotype and database information and/or due to conflicting interpretations. Of the 58 variants, 28 (48.3%) have previously been classified as pathogenic or likely pathogenic in LOVD, HGMD, and/or ClinVar. Ten have been reported as de novo. As de novo coding variants occur at a rate of approximately one per generation across all protein-coding genes [21] and because ACTA1 is so highly conserved, de novo missense variants in *ACTA1* should be considered at least likely pathogenic.

Another limitation of VarSome is that it does not assess potential splice-altering variants using predictive tools such as SpliceAI [13], which has demonstrated very good sensitivity and specificity (> 94% for both) [22, 23]. There are five *ACTA1* VUS with SpliceAI delta scores ≥ 0.2 (the nonclinical “permissive” threshold for splice-altering variants), including three intronic: c.809-10C>A (0.61), c.130-5T>A (0.52), and c.455-7C > A (0.47) and two missense: c.814G>C (p.Glu272Gln) (0.31) and c.818C>G (p.Ser273Trp) (0.2). Of these, only c.455-7C>A is reported in gnomAD (frequency 6.6e − 06).

Taking limitations of VarSome and ACMG criteria into consideration, we have provided our own consensus interpretation of *ACTA1* VUS alongside evidence used to make these conclusions (Supporting Information 3 (Sheet 3.0); VUS analysis). Specific criteria and justification are summarized in the *ACTA1* Variant Classification section.

### 3.3. *ACTA1*Variants in gnomAD

Since its initial release as ExAC in 2016 [24], the gnomAD database has been of great importance for human rare disease genetics. The current v2.1.1 and v3.1 datasets encompass 125,748 exomes and 15,708 genomes [25]. The majority of individuals in gnomAD are older (mean age 54 years) and exclude those with severe pediatric diseases and their relatives [24]. Given the severity of *ACTA1* diseases, the presence of an *ACTA1* variant in gnomAD might therefore be taken as strong evidence to refute pathogenicity for dominant cases, although there are some variants such as c.1099G>A (p.Ala367Thr) that are present in gnomAD but also classified as a VUS in a patient with very late onset disease (52 years) [26], so variant presence in gnomAD may not be completely sufficient to refute dominant disease. There are 602 unique *ACTA1* variants present in gnomAD v2.1.1 and/or v3.1 (Supporting Information 4 (Sheet 4.4)). The majority of these are intronic (286, 47.5%), followed by synonymous (132, 21.9%), missense (79, 13.1%), splice region (42, 7.0%), 3⁣′UTR (28, 4.6%), frameshift (12, 2.0%), stop-gain (9, 1.5%), 5⁣′UTR (5, 0.8%), splice acceptor (4, 0.6%), and splice donor (3, 0.5%) variants.

Notably, *ACTA1* is one of the most missense-intolerant genes in gnomAD v2.1.1 (*Z* = 4.53, *o*/*e* = 0.21; 55 observed/260.9 expected), ranking 164 out of 19,704 genes [27]. That is, only 21% of the expected number of missense variants (based on gene length) have been observed. For context, > 95% of all genes have an *o*/*e* ratio of 0.7 or higher [25]. In contrast, *ACTA1* is relatively tolerant of loss-of-function (LoF) variants (pLI = 0, *o*/*e* = 0.74; 11 observed/14.9 expected). These statistics are concordant with the fact that missense changes at 154/377 positions have been reported as pathogenic, whereas LoF (null) variants are typically associated with recessive disease and are tolerated in heterozygotes [28].

**Figure 3:**
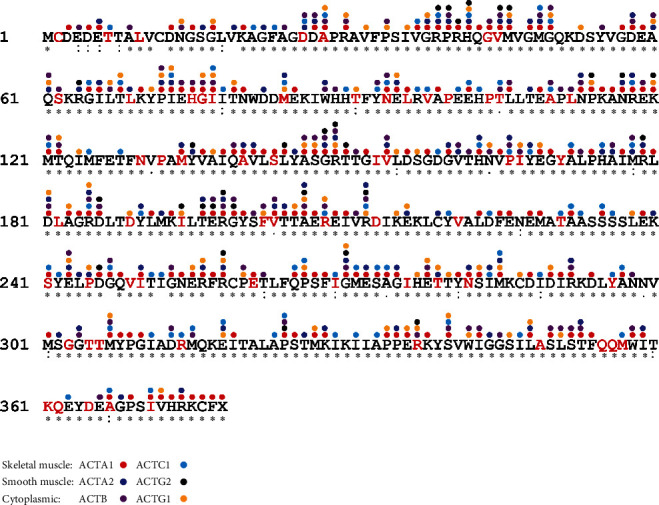
Pathogenic/likely pathogenic mutation sites in ACTA1 and other human actin proteins. Multiple sequence alignment showing completely conserved amino acids (∗), conserved substitutions (:), and semiconserved substitutions (.) across all six human actin proteins: ACTA1 (P68133), ACTC1 (P68032), ACTA2 (P62736), ACTG2 (P63267), ACTB (P60709), and ACTG1 (P65261). Symbols above each amino acid indicate at least one reported pathogenic/likely pathogenic missense variant in the indicated isoform (red: ACTA1; light blue: ACTC1; dark blue: ACTA2; black: ACTG2; purple: ACTB; yellow: ACTG1). For a full list of specific changes, see Supporting Information 4. Amino acid letters in red indicate positions with *ACTA1* missense variants present in gnomAD.

Interestingly, the most common coding change in gnomAD is c.541delG (p.Asp181ThrfsTer11), with 10 reported heterozygotes in South Asian individuals. This variant is a known recessive null variant [28] and represents a likely founder variant within the South Asian population [29]. Twelve other known pathogenic recessive variants are also present in gnomAD as heterozygotes (Supporting Information 4 (Sheet 4.2)). No known pathogenic dominant changes are seen in gnomAD. This is consistent with pathogenic *ACTA1* variants typically causing severe neonatal disease with early lethality [2]. The 79 missense variants in gnomAD affect 65 different residues (Figure 3). Of these 65 positions, only 19 (29%) overlap with the 154 known pathogenic ACTA1 missense sites. The other 46 (71%) affect residues that have not previously been associated with disease.

In sum, *ACTA1* variants in gnomAD can be generally considered to be either (a) not disease-causing or (b) cause recessive disease. LoF variants (Supporting Information 4 (Sheet 4.6)) are likely to represent recessive variants, as might the 72 missense variants not previously associated with disease (Supporting Information 4 (Sheet 4.5)). On account of the exceptionally high missense constraint for *ACTA1*, it would be of interest to test the protein folding of these variants to ascertain whether they represent functional nulls that would cause recessive disease [30].

## 4. *ACTA1* Variant Classification

Although ACMG guidelines provide an excellent framework for consistent variant interpretation across many genes, the original authors acknowledged that “those working in specific disease groups should continue to develop more focused guidance regarding the classification of variants in specific genes given the applicability and weight assigned to certain criteria may vary by gene and disease” [12]. One such example of disease-specific adaptation is ClinGen's recommendations for *MYH7*-associated inherited cardiomyopathies [31]. Further comments and examples are noted in [32].

Given several exceptional characteristics of *ACTA1*, including the incredibly high protein sequence conservation and missense constraint, we believe that such adjustments are advisable for more appropriate classification of *ACTA1* variants. Indeed, when we compiled all known *ACTA1* variants and produced pathogenicity scores based on ACMG guidelines using VarSome, we noted several issues with using such an automated approach for classifying *ACTA1* variants. For example, VarSome does not incorporate some information from LOVD or publications, such as de novo reports and the number of cases in which a variant has been reported. We also determined that the mutation hotspot (PM1) definition was not appropriate for *ACTA1* (which has no specific hotspots or discretely defined functional domains). In our refined model for calculating PM1, the sliding window approach identifies candidate regions between known benign variants within genetic data. These regions are analyzed for their concentration of pathogenic variants. The model employs a dual criterion for PM1 classification: a region with no benign variants and at least one pathogenic variant is considered indicative of likely PM1. Additionally, regions with three or more pathogenic variants also qualify due to the hypothesis that *ACTA1* missense variants are most likely PLP. This method effectively weights the significance of pathogenic variants, particularly in benign-variant-free zones, providing a nuanced approach to classifying VUS and enhancing predictive accuracy in genetic data analysis.

With the above in mind, we reviewed the list of 58 VarSome VUS and reclassified these variants based on additional available information and updated criteria (Supporting Information 3). These classifications are based on the assumption that the variant in question causes dominant disease. However, variants that do not meet the P/LP cutoff using these criteria may still represent recessive variants. Recently, others have also applied ACMG guidelines to reclassify a subset of variants in genes associated with nemaline myopathy [33]. Importantly, we acknowledge that while we have primarily used VarSome for high-throughput analysis of *ACTA1* variants, there are also other tools and initiatives that similarly serve to help standardize variant analysis. We emphasize that it is important for users to understand the limitations of any variant interpretation tool. For example, such tools may not automatically apply gene-specific recommendations. Nevertheless, they have good utility for bulk classification of variants.

Overall, we highlight several criteria and provide some possible adjustments to ACMG guidelines that may improve the interpretation of *ACTA1* variants. These are summarized in Table 2 and should be used in combination with the standard ACMG criteria. We note, however, that most of these suggestions aid the interpretation of dominant missense variants (the most common type of pathogenic *ACTA1* variant). Recessive variants (e.g., frameshift and stop-gain variants) should be interpreted separately. Tools such as SpliceAI may be useful for the interpretation of synonymous or potential splice variants. Such variants may be dominant or recessive, depending on the functional outcome, which should be confirmed by RNA-seq and/or cDNA studies or minigene assays where patient material is unavailable. In general, a SpliceAI score of ≥ 0.2 can be considered to distinguish possible splice-altering variants but only provides moderate evidence. In a clinical setting, a much higher threshold (≥ 0.5) is recommended for the assertion of pathogenicity, albeit at the cost of missing many potential spliceogenic variants [35]. Recommendations on how to classify putative splice-altering variants using the ACMG/AMP framework have been provided by the ClinGen Sequence Variant Interpretation Splicing Subgroup [35].

Based on our VUS reclassification (Supporting Information 3) and the addition of novel variant reports (Supporting Information 2 (Sheets 2.10 and 2.11)), there are 447 P/LP, 13 VUS, and 147 B/LB *ACTA1* variants (607 in total). As noted above, the final variants (following analysis) are collated in Supporting Information 2 (Sheet 2.0). This table has been formatted to make it simpler to search for variants. For full, detailed information on individual variants (such as references and number of reported cases), readers should examine Supporting Information 2 (Sheet 2.1) (full variant table) and Supporting Information 2 (Sheets 2.10 and 2.11) (extra variants from February 2023 to August 2024).

### 4.1. Other Important Considerations for ACTA1 Variant Interpretation

#### 4.1.1. Mosaicism

Since most patients with *ACTA1* variants do not reach reproductive age, there is a high frequency of de novo variants in *ACTA1* patients [2]. For other patients, the variant is inherited from an apparently unaffected or mildly affected parent that has mosaicism for the variant. Mosaicism for *ACTA1* was first described in 1999 by Nowak et al. [1], and multiple other instances have since been described (e.g., [36]; Supporting Information 4 (Sheet 4.1)), including one case of gonadal mosaicism masquerading as autosomal recessive nemaline myopathy [37].

Mosaic cases continue to be found regularly in both diagnostic and research laboratories [38]. We have recently identified a mosaic family in which the mother contains the c.115C>G p.(Arg39Gly) variant at an allele balance of 0.09. This variant has not been previously reported, although it is a known pathogenic variant in *ACTA2* [39]. The mother had a very mild disease, whereas the proband was severely affected and stillborn. Such cases exemplify the known correlation between ACTA1 mutant-to-wildtype protein ratio and disease severity, whereby mosaics are typically less severely affected due to comparatively lower levels of the mutant protein.

Instances of *ACTA1* mosaicism may be missed by standard diagnostics such as Sanger sequencing but can be identified by ultradeep sequencing [40, 41]. The level of mosaicism may vary between tissues [40]. For example, Lornage et al. identified *ACTA1* mosaicism in muscle from patients (9%–12% of reads) that was barely detectable in blood DNA [42]. Importantly, this report also provided the first evidence of congenital myopathy with asymmetric disease caused by mosaicism. The asymmetry and tissue-disparate variant frequencies were hypothesized to be caused by postzygotic mutation of *ACTA1* after left-right determination. However, this does not necessarily explain why variant-containing cells and pathology are still present on both sides of the body. Very recently, Lehtokari et al. described a case series of four mosaic individuals with recurrent c.739G>A and c.739G>C (p.Gly247Arg) variants that also exhibit asymmetry [38].

Both somatic and germline mosaicism must be considered as possible modes of inheritance for *ACTA1* disease families. If an *ACTA1*-related phenotype is present but no pathogenic variant is initially identified, it would be worthwhile to investigate possible *ACTA1* mosaicism (e.g., reduce stringency of variant filtering parameters). It may be advisable to counsel a couple and manage subsequent pregnancies as if a parent is known to be heterozygous for the variant.

#### 4.1.2. Pathogenicity in Other Actins

Others have recently highlighted the utility of integrating gene family information and conservation for variant interpretation [43, 44]. Thus, we propose that known pathogenic changes in other actins could be used to assert the pathogenicity of novel *ACTA1* variants, particularly where the variant is absent from population databases such as gnomAD.

ACTA1 is one member of the actin protein family—a family of highly conserved proteins with diverse functions including maintenance of the cytoskeleton, cell motility, and muscle contraction [45]. The other five vertebrate actin isoforms are alpha-cardiac (ACTC1), alpha-smooth muscle (ACTA2), gamma-smooth muscle (ACTG2), beta-cytoplasmic (ACTB), and gamma-cytoplasmic (ACTG1) [46]. In general, the cytoplasmic actins (ACTB and ACTG1) are ubiquitously expressed, and ACTA2 and ACTG2 are found in smooth muscle. In adults, the striated muscle actins ACTA1 and ACTC1 are expressed most highly in skeletal and cardiac muscle, respectively [47]. Importantly, ACTC1 is the main skeletal actin in skeletal muscle development until ~27–28 weeks when ACTA1 becomes the dominant isoform [48], and in the adult heart, ACTA1 and ACTC1 are coexpressed, with ACTA1 making up approximately 20% of total actin [49].

**Figure 4:**
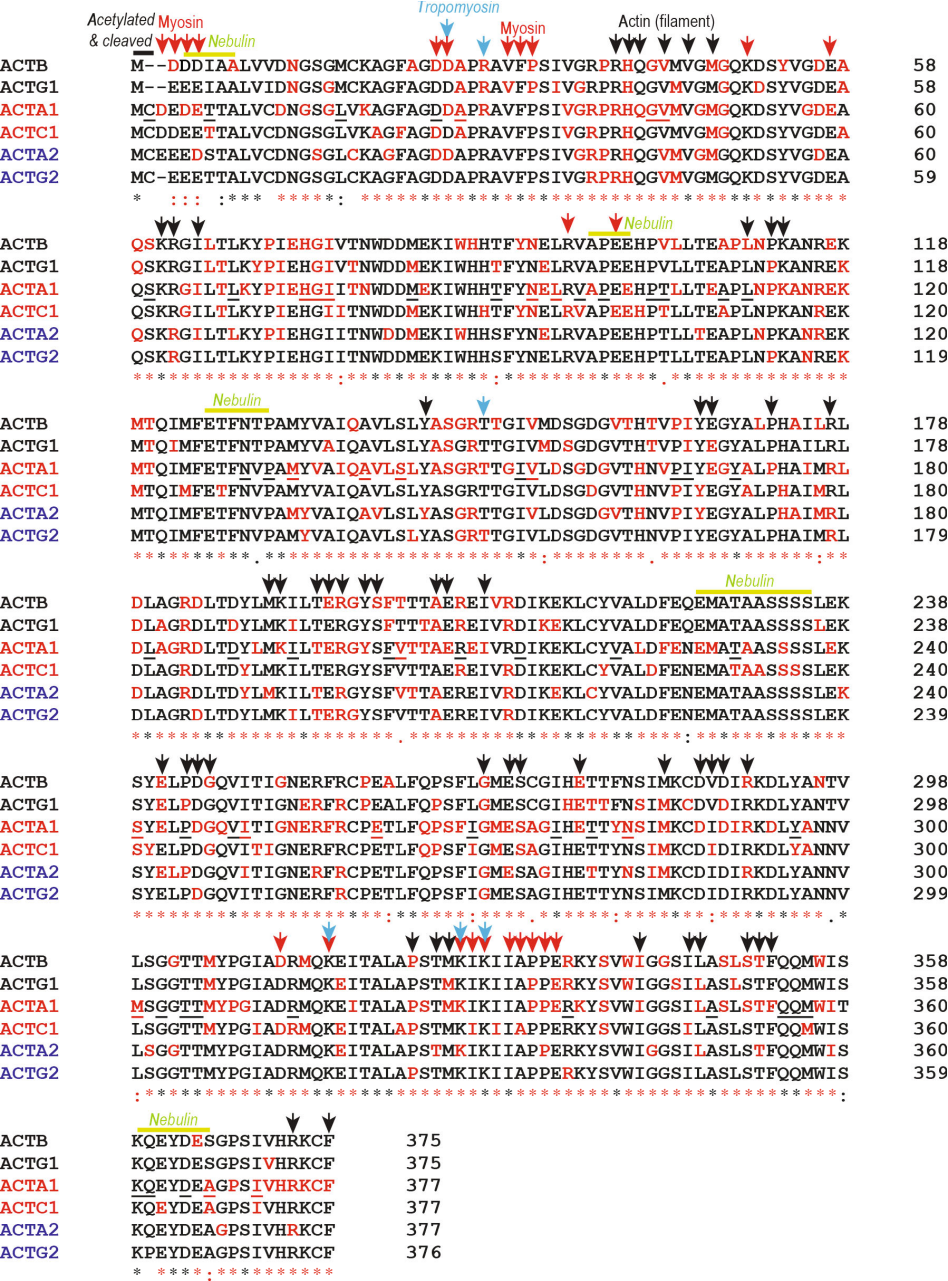
Multiple sequence alignments of all human actin proteins. Pathogenic/likely pathogenic missense changes (from HGMD, LOVD, and ClinVar) are indicated in red. Underlined residues in ACTA1 only indicate missense changes reported in gnomAD. Annotations mark cleaved N-terminal residues (black line) and interactions with myosin (red arrows), actin filament (black arrows), tropomyosin (blue arrows), and nebulin (green lines), as annotated in [34] in greater detail). UniProt sequence IDs: ACTA1 (P68133), ACTC1 (P68032), ACTA2 (P62736), ACTG2 (P63267), ACTB (P60709), and ACTG1 (P65261). Skeletal actins (ACTA1 and ACTC1) are labelled in red, smooth muscle actins (ACTA2 and ACTG2) in blue, and cytoplasmic actins (ACTB and ACTG1) in black.

At the amino acid level, all mammalian actin isoforms are highly similar—no isoform shares less than 93% homology with any other isoform (Figure 4) [50]. Strikingly, genomic sequence and 3D protein model comparisons show extremely high conservation of alpha-skeletal actin between vertebrates and muscle-like actins in plants and yeast [45]. This is particularly noteworthy given their evolutionary distance. The high degree of amino acid sequence conservation across species and isoforms suggests that most protein-altering variants in actin are likely to be pathogenic and that pathogenic variants in one isoform are highly likely to be pathogenic in other isoforms.

As of 2012, variants in all actin isoforms have been implicated in disease. A summary of all known disease-associated actin variants in HGMD, LOVD, and ClinVar is shown in Figures 3 and 4. Remarkably, these collectively involve missense changes at 345/377 (91.5%) amino acid residues (Figure 4), supporting the view that every amino acid residue in the actins will ultimately be associated with human disease. This includes 782 reported missense changes; 562 minus duplicates reported in multiple actins. ACTA1 has the most disease-associated changes at 154/377 (40.8%) residues (plus the stop codon), followed by ACTB (100/375; 26.6%), ACTA2 (86/377; 22.8%), ACTC1 (79/377; 21.0%), ACTG1 (78/375; 21.0%), and ACTG2 (30/376 = 8.0%). Variants with major effects on actin function are likely incompatible with life. We have plotted all P/LP amino acid changes reported in human actins, along with brief notes on phenotype/s and sources (Supporting Information 5). This interactive data file is likely to be useful for readers to assess the pathogenicity of novel actin variants.

As a complement to our analyses, Parker, Baboolal, and Peckham have recently reviewed variants across the different actins and their role in disease, with a particular focus on functional and mechanistic insights [34]. Collectively, these datasets may be useful to efficiently and comprehensively assess novel *ACTA1* variants.

## 5. Phenotypic Expansions and Genotype–Phenotype Correlations

### 5.1. Phenotypic Summary and Expansions

**Figure 5:**
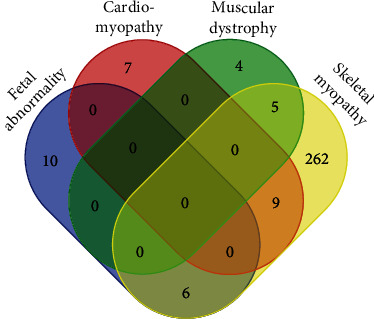
Broad disease categories of pathogenic/likely pathogenic *ACTA1* variants. Venn diagram of all pathogenic/likely pathogenic *ACTA1* variants with reported phenotypes (in LOVD and/or HGMD). Venn diagram constructed using https://bioinformatics.psb.ugent.be/webtools/Venn/.

The spectrum of disease caused by variants in *ACTA1* is broad. In total, there are 20 specific phenotypes associated with *ACTA1* variants (Supporting Information 1 (Table S2) and Supporting Information 2 (Sheet 2.6)). For this reason, diseases caused by *ACTA1* variants are broadly termed “*ACTA1* disease” or actinopathies [51]. There have been several additions to the *ACTA1* disease spectrum since the 2009 mutation update, both novel phenotypes and expanded phenotypes with novel comorbidities (Supporting Information 2 (Sheet 2.6)), as summarized in Supporting Information 1 (Tables S4, S5, and S6). An overview of the phenotypic overlap of individual variants is shown in Figure 5 (variants listed in Supporting Information 1 (Table S3)). Several noteworthy new phenotypes are discussed below.

The majority of P/LP variants (74%) cause nemaline myopathy (NEM3, now CMYP2A; OMIM#161800), followed by congenital fibre type disproportion (CFTD, now CMYP2C; OMIM #620278) at 7.2%, and intranuclear rod myopathy (IRM) at 4.3% (Supporting Information 1). Sixteen variants (5.3%) have been reported to cause fetal abnormalities, such as arthrogryposis and/or fetal akinesia. Sixteen variants (5.3%) have been associated with cardiomyopathies, most commonly dilated cardiomyopathy (*n* = 11), followed by hypertrophic cardiomyopathy (*n* = 5).

*ACTA1*-associated cardiomyopathies have historically presented alongside skeletal myopathy [52]. The first report of an *ACTA1* variant causing cardiomyopathy without clinical skeletal myopathy was reported in 2018 [53]. This variant (p.Arg256His) was novel at the time, and it should be noted that this submission used prior published evidence to support pathogenicity and incorrectly stated the variant has been characterized in prior reports of NEM [1]. The p.Arg256His variant reported by Nowak et al. uses legacy nomenclature and should actually be p.Arg258His according to HGVS. This provides an excellent example of where legacy nomenclature can confuse and confound the interpretation of some *ACTA1* variants. However, since the 2018 report, the p.Arg256His variant has been reported as pathogenic in ClinVar and is supported by additional pathogenic missense variants at the same amino acid position (p.Arg256Leu and p.Arg256Gly, Supporting Information 2 (Sheet 2.1)).

### 5.2. Distal Myopathy

The first cases of *ACTA1* disease with distinct distal involvement were reported in 2015 [54]. This report described the largest actinopathy pedigree to date, comprising six generations and 33 affected individuals with scapuloperoneal myopathy caused by a c.591C>A (p.Glu197Asp) variant in *ACTA1*. Affected individuals showed clinical and morphological features distinctive from other actinopathies, including scapulo-humeral-peroneal distribution with striking upper extremity predilection in some individuals, progressive but variable disease course, and sparing of respiratory muscles until advanced stages of the disease. Muscle biopsy showed no signs of nemaline rods but showed lobulated or trabeculated fibres in advanced biopsies, which have not been previously reported.

Since 2015, an additional three families with distal myopathy have been reported: an unrelated family with scapuloperoneal myopathy and the same p.Glu197Asp mutation but with abundant nemaline rods [55], a family with early-onset distal myopathy with preferential involvement of anterior leg muscles and finger flexors and a novel p.Gly253Arg substitution [7], and a family with predominant finger flexor weakness caused by a p.Gly50Asp mutation [56]. The latter is one of only two reports that show rimmed vacuoles in *ACTA1*-myopathy (the other is Sewry et al. [57]). Together, these reports clearly demonstrate that ACTA1 variants can present with primary distal involvement.

### 5.3. Additional Noteworthy Cases and Phenotypes

Several new pathologies and/or copathologies have been associated with *ACTA1* variants since the last review in 2009 (Supporting Information 1 (Tables S4, S5, and S6)). In 2012, Jain et al. described a NEM patient with hypercontractility instead of typical weakness and hypotonia caused by a p.Lys328Asn mutation that increases the activated state of the thin filament [58]. Hung et al. describe a patient with a p.Met49Val change and cap myopathy characterized by sarcolemally-located caps immunopositive for *α*-actinin and actin [59]. Donkervoort et al. and Schuelke et al. separately describe two changes (p.Asn94Lys and p.Phe92del) and cytoplasmic body myopathy, with no evidence of nemaline rods [60, 61]. Two reports separately describe cases of recessive [62] and dominant [63] myofibrillar myopathy caused by p.Ile250Thr and p.146_147dupAlaSer variants, respectively. Cerino et al. noted a case of a rimmed vacuolar myopathy in a *GNE*-negative cohort [64], although there is no convincing evidence for the identified *ACTA1* variant (p.Ala146Val) being causative, and this change has since been removed from HGMD as being disease-causing. However, an independent report has also linked an *ACTA1* variant (p.Gly50Asp) with a rimmed vacuolar myopathy phenotype. In this case, the patient was initially misdiagnosed with “inclusion body myositis” sans inflammatory changes based on the pattern of weakness observed [56]. Collectively, these cases highlight particularly interesting and unique *ACTA1* disease phenotypes.

### 5.4. Genotype–Phenotype Correlations

There are no obvious correlations between pathological changes and molecular findings in *ACTA1*. In the most comprehensive study to date, Feng and Marston concluded that the lack of apparent correlations reflects our limited knowledge about actin structure–function relationships [6]. They suggest that mutations are likely to affect several functions together, making it virtually impossible to unravel a clear genotype-phenotype relationship. Recent new structural insights into actin [65] may help decipher genotype-phenotype correlations.

A major complication in assigning specific phenotype–genotype correlations for *ACTA1* disease is that there is a significant spread in clinical manifestations and severity, even for the same variant. For example, the p.His40Tyr substitution is known to cause severe NEM and death within two months [1] but has also been identified in a patient with typical NEM who was alive at 42 years [36]. Similarly, Hutchison et al. describe a four-generation family with five NEM-affected individuals with the same ACTA1 p.Val163Met mutation but variable presentation [66]. Although affected individuals had broadly similar phenotypes, muscle pathology varied significantly; abundant intranuclear rods were found in two cases (grandfather and infant), but another affected family member (mother) did not show any signs of intranuclear or sarcoplasmic rods in clinically involved muscle following careful examination.

Although it is still a major question as to why such clinical and histological variation exists (especially between individuals with the same variant), we include some known variables and speculations below:

1. *Nemaline rods are not always seen on biopsy*. It is well documented that the presence of nemaline bodies can vary depending on the muscle biopsy as well as the sampling location within the biopsy [67]. In many cases, additional biopsies are needed to show nemaline bodies. This is a common reason for histological variability in NEM cases.
2. *Modifier genes.* The presence of modifying factors, such as additional variant/s in other muscle genes (that alone may have little to no effect), may reduce or enhance disease pathology by exacerbating the effect of the pathogenic ACTA1 protein or by causing increased expression of the mutant *ACTA1* gene/transcript.
3. *Relative levels of mutant skeletal muscle actin and dilution by ACTC1.* In recessive *ACTA1*-disease, retention of ACTC1 (fetal skeletal alpha-actin) is known to reduce disease severity [28]. More recently, higher levels of ACTC1 in skeletal muscle have also been correlated with milder phenotypes in dominant disease [68]. The timing and dosage of ACTC1-to-ACTA1 isoform switching during the fetal-to-adult transition may similarly affect the severity, whereby certain individuals may be more likely to develop severe pathology if the timing of the ACTA1 transition occurs during a critical developmental point (due to less ACTC1 dilution).
4. *Some variants are more likely to cause severe pathology based on their location within the protein.* Many *ACTA1* variants reported to cause severe disease and/or fetal abnormalities also cause severe disease in additional cases (e.g., Glu74Lys, Arg185Cys, Glu261Val, Asn282Lys, Asp288Gly, and Val372Phe), whereas variants documented as causing more mild pathology are more likely to produce a wider spectrum of disease (e.g., Met134Val, Val136Ala, and Gln248Arg). Of relevance, Chong et al. recently described for the first time several *ACTC1* variants that cause severe skeletal muscle pathology, that is, distal arthrogryposis [69]. Importantly, they note that homologous variants in *ACTA1* also cause severe pathology and tend to disrupt the native structure of the regions of actin most associated with protein–protein interactions (SD2 and D-loop). Therefore, although it can be difficult to predict severity based on the variant alone, many variants do tend to have consistent pathology. With further reports, such correlations will become easier to understand. Ultimately, we hope that our collation of ACTA1 and other actin variants highlights which residues are more critical for function and therefore more likely to cause severe disease.

In sum, although there exists significant variation in pathology across ACTA1 variants, prior reports are still very valuable to help inform expected phenotypes. Further, a variant should not be discounted as pathogenic if disease manifestations differ from other prior reports for the same variant. Finally, we note that *ACTA1* should be considered a possible disease gene candidate in a wide range of congenital myopathies, even if the genotype–phenotype correlation has not yet been established.

## 6. Disease Mechanisms

The majority of pathogenic variants in *ACTA1* are dominant missense substitutions that act by altering actin polymerisation dynamics, stability, and interactions with actin-binding proteins [6, 36]. In contrast, recessive variants are LoF variants or missense changes that prevent normal folding of the actin monomer, leading to a functionally null protein [30]. The pathomechanisms of various ACTA1 substitutions have been dissected and reviewed previously [51, 70]. Therefore, here, we primarily discuss interesting mechanisms and insights that have more recently emerged.

### 6.1. Actin Filament Conformation and/or Polymerisation into F-Actin

Detailed structure-function studies have provided important mechanistic insights into how ACTA1 mutations drive disease. One well-studied example is the Ser350Leu substitution, associated with actin filament aggregate myopathy [71]. Recent 3D modelling suggests that this mutation generates an extra *α*-helix, which disrupts *α*-actinin binding, and consequently, F-actins are not able to bind properly to the Z-line [72]. Interaction with gelsolin is also disrupted which destabilizes the F-actin structure. Increased hydrophobicity due to the mutation leads to aggregations of F-actin in myofibres. This aggregation subsequently disrupts interaction with myosin rather than a direct disruption of actomyosin linkages, as was first hypothesized.

### 6.2. Disruption of Sarcomere Structure, Function, and Dynamics

Sarcomeric defects typically result when mutant proteins incorporate into sarcomeric structures in significant amounts without disrupting muscle architecture [73]. Work performed on muscle biopsies from 14 *ACTA1* patients showed shortened thin filaments and decreased force generation in a subset of patients with specific mutations [74]. Further, significantly lower tension and stiffness in myofibres were found to contribute to muscle weakness [75], which was not specifically due to the shortened thin filaments but rather a reduced number of myosin heads binding to actin. Together, these studies provide additional insights into the mechanisms of ACTA1 mutations and highlight that the precise mode of action is often complex and mutation-dependent.

### 6.3. Novel and Underconsidered Disease Mechanisms—Nuclear Function and PTM

Although the function of ACTA1 in the nucleus is unclear, it is known that short actin polymers are present inside the nuclei of myoblasts and directly interact with emerin and lamin in myotube nuclei [76]. Actin is normally present in the nucleus only in trace amounts, owing to a nuclear export signal encoded by residues 170–181 and 211–222 in cytoskeletal actins (172–183 and 213–224 in ACTA1) [77]. Ilkovski et al. [78] showed that *ACTA1* variants identified in patients with intranuclear rods (e.g., Val165Leu, Val165Met, and Arg185Gly) tended to also produce intranuclear rods in culture [78]. Therefore, there does seem to be a correlation between the variant and the likelihood of producing intranuclear rods. They proposed these variants disrupted nuclear export signals. Later, Domazetovska et al. used live-cell imaging to demonstrate that the nuclear aggregates of actin form within the nuclear compartment rather than entering the nucleus after formation in the cytoplasm [79]. They showed that the organization of actin within these aggregates is influenced by the binding of alpha-actinin (the principal protein of the Z-disc and cytoplasmic nemaline bodies) and that alpha-actinin is also normally present in the nucleus of muscle and nonmuscle cells. They also proposed the variants associated with intranuclear rods increase ACTA1 trafficking into the nucleus. For some variants (e.g., Val165Met), the sequestration of sarcomeric and Z-line proteins into intranuclear aggregates correlates with muscle regeneration which has been suggested may explain more mild disease [36, 66, 78, 80].

Recently, Ross et al. revealed novel pathological defects in skeletal muscle nuclei of mouse models and patients with NEM (ACTA1 Glu6Lys, Tyr281His, Phe226Leu, Thr79Ala, and Tyr281His) [81]. Defects included irregular spacing of nuclei, disrupted nuclear envelope, altered chromatin arrangement, and disorganization of the cortical cytoskeleton. They proposed that such defects would contribute to a range of disease features including broad transcriptional alterations and hindered myofibre growth [82, 83], myofibril disarray, and altered mechanical properties of myofibres [84]. More recently, Labasse et al. reported enlarged perinuclear space in several *ACTA1* patients with nemaline myopathy [68]. They suggest that *ACTA1* variants or ACTA1 substitutions may directly or indirectly impact the function of F-actin as a molecular linker and that aberrant nuclear envelope architecture may interfere with gene expression as in nuclear envelopathies [85]. Overall, these findings extend the array of mechanisms and phenotypes that can be studied when assessing the pathogenicity of *ACTA1* variants.

More than 140 post-translational modifications have been described in eukaryotic actins across 94 different side chains (reviewed in [86]). Alterations to these PTMs have been shown to affect actin function by inhibiting actin polymerisation, ATP binding, and ATPase activity, frequently leading to F-actin depolymerisation and aggregation [87–89]. SUMOylation of residues Lys68 and Lys284 is speculated to regulate nuclear trafficking and actin structure [90, 91]. Thus, when assessing the pathogenicity or mechanism of specific missense changes in ACTA1, it may be useful to note whether such changes could impact critical PTMs.

## 7. Preclinical Models of *ACTA1* Myopathy and Potential Treatment Strategies

There are no cures or treatments for *ACTA1* myopathy; thus, there is an urgent unmet need to develop effective treatments capable of addressing the underlying disease mechanisms. Some promising treatments have been tested preclinically, but so far, none have progressed to clinical trials.

### 7.1. Cell and Animal Models of ACTA1 Myopathy

Three mouse models of *ACTA1* myopathy exist. The features of each model have been well summarized by Sewry, Laitila, and Wallgren-Pettersson [29]. Briefly, the *Acta1* knockout line (*Acta1*-KO) is a model of recessive *ACTA1* myopathy [92], and homozygous *Acta1*-KO mice typically die by 9 days postnatal. The other two murine models both harbour dominant pathogenic variants: a hemizygous knock-in model of the human H40Y (legacy nomenclature) variant (*Acta1*^H40Y^; [93]) which is considered a moderate–severe model of disease [29], and a transgenic model of the human D286G (legacy nomenclature) variant (Tg(*ACTA1*)^D286G^; [19]). These models have undoubtedly been and continue to be valuable for the investigation of pathomechanisms and treatments but do have some limitations.

The Tg(*ACTA1*)^D286G^ line has been shown to model various features of human disease including muscle weakness, myofibrillar disruption, and the presence of nemaline rods [19, 94, 95]. However, in our hands, we have observed phenotypic drift of this line over time (unpublished), suggesting that the transgene expression may be unstable [96, 97]. This proposed instability may lead to colony-specific phenotypic variation. For example, in 2018, Tinklenberg et al. replicated the original findings that Tg(*ACTA1*)^D286G^ mice displayed impaired rotarod and voluntary running wheel activity, and histopathological abnormalities [98] but were unable to replicate the originally reported weight deficit [19]. As a knock-in model, the *Acta1*^H40Y^ line is not subject to transgene instability; however, the males of this line have a high incidence of mortality due to bladder outlet obstruction [99]. These points do not preclude these models from being used to test and develop treatments but should be taken into consideration.

Given the diversity of *ACTA1* variants and their downstream pathobiological effects and mechanisms, we propose that additional models are needed to facilitate effective screening of new treatments in a range of mutational contexts. The Mutagenetix database (Beutler et al.; https://mutagenetix.utsouthwestern.edu) catalogues mutant mice generated randomly by ENU germline mutagenesis [100] and contains 11 records for mice harbouring *Acta1* variants (Table 3, data current at 7 December 2023). It should be noted that G1 mice generated by ENU (N-ethyl-N-nitrosourea) mutagenesis are estimated to carry an average of 30–40 coding or splice-site altering variants [101], and these mice have not yet been specifically analyzed for muscle-related phenotypes. Thus, the disease assertions of these variants remain to be determined. However, two mouse lines are heterozygous for known pathogenic variants (Table 3); line R9651 harbours an autosomal recessive p.Tyr220Ter variant (unpublished), while the Z1177 line harbours a p.G44V variant which is autosomal dominant and associated with mild disease [36]. In addition, four of the 11 mice harbour amino acid changes which have not been previously reported but occur at positions which harbour likely pathogenic mutations in patients (Table 3). Therefore, some of these mice may represent new models of *ACTA1* disease. Of note, we have previously investigated the R0090 strain harbouring a p.N14S variant. Heterozygous *Acta1*^N14S^ mice did not have an overt phenotype, but homozygous mice exhibited early postnatal lethality suggestive of recessive disease (unpublished). Further, our attempts to generate new *Acta1* mouse models by CRISPR gene editing were also unsuccessful, likely due to many *Acta1* variants producing severe phenotypes incompatible with breeding (unpublished).

In addition to mouse models, patient-derived cell models are likely to be a valuable tool. We have generated iPSC lines from three dominant *ACTA1* patients (c.515C>A (p.Ala172Glu); c.541G>A (p.Asp179Asn); and c.553C>A (p.Arg183Ser)) [102–104] and one recessive patient (c.121C>T (p.Arg39Ter)) [105]. All three of these variants are described using legacy nomenclature, so as to match the original reports that described these individuals. We have also generated a dominant *ACTA1* patient (p.Gly148Asp) iPSC line with a matched corrected isogenic control line [106]. Finally, Kim et al. have produced an iPSC line from an *ACTA1* (c.1029C>A, p.Ile343Met) patient with hypertrophic cardiomyopathy [107].

Protocols for the differentiation of iPSCs to skeletal muscle have been refined to enable the production of large numbers of skeletal muscle progenitors that can be differentiated to myotubes with high fusion index [108–110]. Recently, Gartz et al. generated CRISPR-edited *ACTA1*^H40Y^ lines that display reduced ATP levels and mitochondrial membrane potential following differentiation to skeletal muscle myotubes [111]. Mitochondrial defects (including reduced ATP levels but not decreased membrane potential) were also observed in the *ACTA1^H40Y^* mouse model [112], and Mitochondrial Complex I deficiency has previously been reported in an *ACTA1* patient [113]. Together, this suggests that iPSC-derived muscle cells may be a useful tool for modelling some *ACTA1* myopathy phenotypes. Thus, the *ACTA1* iPSC lines generated to date may be a useful resource for testing treatments; however, their ability to model quantitative disease-relevant phenotypes remains to be fully established. With this in mind, 3D culture systems such as the “Mantarray” may be useful for assessing clinically relevant functional deficits such as contractile force [114]. 3D systems will also be essential to allow sufficient maturation of cultures so that they accurately recapitulate muscle dynamics, including ACTA1 expression.

Several zebrafish models of *ACTA1* myopathy, including both dominant (Tg(*ACTA1*^D286G-eGFP^)) and recessive (*Actc1b* morphants), have been established and used to model the mechanisms of nemaline body formation [115] and test treatments [116]. Zebrafish have many advantages as an in vivo model due to their large reproductive capacity, relatively short life cycle, small size, and optical clarity [117]. Further, muscle functional readouts (force) can be obtained as early as 2 days postfertilisation using quantitative tests of swimming performance [118]. These properties make zebrafish suitable for conducting high-throughput screens.

Collectively, these animal and cell models could be used in various combinations as part of a treatment testing pipeline to ensure that potential new treatments are rigorously tested at the preclinical level.

### 7.2. Current State of Preclinical Treatments

Therapeutic interventions for actinopathies were recently reviewed by Gineste and Laporte [119]. To summarize, L-tyrosine supplementation [116, 120] was not conclusively shown to improve muscle weakness in *Acta1* models. Myostatin inhibition produced nuanced results, improving life-span but not muscle weakness in the *Acta1*^H40Y^ model [99] and improving muscle weight and absolute force in the Tg(*ACTA1*)^D286G^ model without improvement in other disease-associated phenotypes (e.g., specific force, running wheel performance) [98]. *MYL4* gene therapy may be a promising approach with therapeutic potential for ACTA1 variants that impair myosin binding [81, 82]. Further investigation of this strategy is required to ensure its utility for a range of *ACTA1* variants and ascertain whether similar benefits are achieved following systemic injection. A fast skeletal muscle troponin activator, *tirasemtiv*, has also been shown to augment thin filament sensitivity to calcium and improve muscle contractility in the *Acta1*^H40Y^ model and in patient muscle tissues [121].

Gineste and Laporte [119] also reviewed preclinical treatments for congenital myopathies more broadly and highlighted the potential of traditional gene therapies (e.g., AAV-mediated cDNA delivery) and oligonucleotide-based therapeutics (e.g., antisense oligonucleotides [ASOs], siRNA) for this group of diseases [119]. As yet, there have been no published reports testing these treatment options in the context of *ACTA1* myopathy. However, given that the ratio of wildtype to pathogenic protein is a critical factor in *ACTA1* disease severity for dominant variants [122], ASOs/siRNAs which selectively reduce the amount of pathogenic *ACTA1* mRNA are likely to be viable treatment options. In addition, overexpression of cardiac alpha-actin (*ACTC1*) in the Tg(*ACTA1*)^D286G^ model was also shown to have significant therapeutic potential [123], providing support for further investigation of gene therapy in this context.

Overall, there is an urgent need for further investment in complex models of skeletal muscle to advance preclinical testing of novel therapeutics and facilitate their translation.

## 8. Conclusion

Based on the collation and reclassification of novel and previously described variants, we report a total of 447 P/LP variants in *ACTA1* and an additional 11 that remain VUS. Overall, this article and the supporting information provided should serve as a valuable resource for the effective interpretation of *ACTA1* variants and thus have important diagnostic utility.

## Figures and Tables

**Table 1:** Distribution of reported pathogenic/likely pathogenic variants across the *ACTA1* gene (HGMD, LOVD, and ClinVar).

	**Length (bp)**	**# coded AA (% of total)**	**Missense (# unique positions)**	**Stop-gain (# unique positions)**	**Frameshift (# unique positions)**	**Other**	**Total**	**% of total variants**
Exon 1	91	0	0	0	0	0	0	0%
Intron 1	876					0	0	0%
Exon 2	141	43 (11%)	25 (17)	2 (2)	1 (1)	0	28	8.4%
Intron 2	106					0	0	0%
Exon 3	325	108.3 (29%)	94 (53)	3 (3)	9 (9)	1	107	31.9%
Intron 3	124					1	1	0.3%
Exon 4	162	54 (14%)	55 (29)	1 (1)	3 (3)	0	59	17.6%
Intron 4	84					3	3	0.9%
Exon 5	192	64 (17%)	51 (29)	4 (4)	1 (1)	0	56	16.7%
Intron 5	91					3	3	0.9%
Exon 6	182	60.6 (16%)	33 (26)	1 (1)	1 (1)	1	36	10.7%
Intron 6	78					1	1	0.3%
Exon 7	398	47 (12%)	46 (22)	1 (1)	1 (1)	1	49	14.6%
Total		377 AA	304 (154)	12 (12)	16 (16)	11	343	

Abbreviation: AA = amino acid.

**Table 2:** Modified ACMG criteria to consider for the interpretation of *ACTA1* variants (in addition to standard criteria).

**Criteria**	**Evidence classification**	**Reference dataset**
1. Does the phenotype/s match with prior cases of *ACTA1* disease?	If yes ➔ candidate variant. If phenotype highly specific for *ACTA1* (e.g., NEM) ➔ PP4 If no ➔ could the phenotype conceivably be caused by an *ACTA1* variant?	Supporting Information 1 (Table S2) (phenotypes) and Supporting Information 2 (Sheet 2.6) (variants/phenotypes)
2. Has the variant been reported de novo (current case, or previous case)?	If yes ➔ PS2 (de novo reports are almost certainly pathogenic based on prior reports). If no ➔ does the variant segregate with disease? ➔ if yes: PP1_supporting, PP1_moderate, PP1_strong (depending on number of family members) If suspected yes (unconfirmed) ➔ if the variant is expected to be de novo (without paternity and maternity confirmed) ➔ PM6	Supporting Information 2 (Sheet 2.1) (“de novo” column)
3. Is the variant a stop-loss variant?	If yes ➔ PVS1; expected pathogenic—All single nucleotide changes that cause stop loss (aside from indels) result in the inclusion of 47 additional AA at the C-terminus and are pathogenic [16].	
4. Is the variant a null variant (nonsense, frameshift, canonical ±1 or 2 splice sites, initiation codon, single or multiexon deletion)?	If yes ➔ PVS1 if recessive; look for a second hit on the other allele. Or, if the variant found in Exon 7 or 3⁣′ end of Exon 6, may escape NMD and lead to dominant disease; possible PVS1.	
5. Is the variant a potential splice variant?	*SpliceAI* *score* ≥ 0.5 ➔ PP3 (if resulting outcome of change/s could feasibly be pathogenic)	Use SpliceAI or similar to assess likely splice-altering event ➔ confirm by RNA-seq/cDNA studies or minigene assay where patient material is not available
6. Is the variant a missense variant?	If yes ➔ PP2 (ACTA1 has incredibly high missense constraint; only a single confirmed benign missense variant at poorly conserved position).	
7. Do multiple in silico predictions support pathogenicity?	*CADD* *score* < 20 and AlphaMissense = likely benign/ambiguous ➔ Not PP3 (variant is likely benign) *CADD* *score* ≥ 20 and AlphaMissense = likely pathogenic ➔ PP3 Note: various tools are appropriate here; VarSome and other tools will often consider multiple predictions to classify as PP3.	Assess variant using VarSome and/or CADD (VarSome includes CADD analysis; *CADD* *scores* ≥ 20 are a generally good indicator of pathogenicity of *ACTA1* variants, *score* > 30 = *high* *confidence*)
8. Has the same variant/AA change in ACTA1 been reported P/LP in HGMD, LOVD, and/or ClinVar?	If yes ➔ PP5 (HGMD and LOVD reports typically provide higher confidence as they provide sufficient phenotypic description whereas most ClinVar reports do not).	Supporting Information 2 (Sheet 2.1) and Supporting Information 5 (Sheets 5.1 (all actins), 5.2 (ACTA1 and ACTC1), and 5.3 (ACTA1 only)). Number of reports can also add further confidence
9. Has a different AA change at the same residue in ACTA1 been reported P/LP in HGMD, LOVD, and/or ClinVar?	If yes ➔ PM5 (another missense change at the same residue provides evidence that the position is intolerant of change).	Supporting Information 2 (Sheet 2.1) and Supporting Information 5 (Sheets 5.1 (all actins), 5.2 (ACTA1 and ACTC1), and 5.3 (ACTA1 only)). Number of reports/different AA changes can also add further confidence
10. Has the same variant/AA change been seen in gnomAD?	If no ➔ PM2 If yes ➔ not PM2, possible BS1 (if gnomAD report is in an older individual, e.g., > 50 years, variant is likely BS1) For recessive cases: no homozygotes ➔ PM2	Supporting Information 4 (Sheets 4.4 (all), 4.5 (missense), and 4.6 (frameshift/stop-gain))
11. Has the same variant/AA change been reported B/LB?	If yes ➔ BS2	Supporting Information 2 (Sheets 2.1 (all variants) and 2.5 (B/LB variants))
12. Is the affected AA position completely conserved across all actins?	If yes ➔ PM1? If no ➔ not PM1? (based on fact that (i) ACTA1 has no hotspots, (ii) is very highly conserved across species and actins, and (iii) has extremely high missense constraint and only one reported benign missense variant ➔ a completely conserved residue with at least one reported P/LP change and no benign variants could be considered a “hotspot” in ACTA1).	Figures 3 and 4 and Supporting Information 5 (Sheet 5.1)
13. Has the same variant/AA change been reported P/LP in any other actin?	If yes ➔ PS1? (based on the fact, actins are highly conserved; highest confidence for ACTC1, then ACTA2/ACTG2, then ACTB/ACTG1).	Supporting Information 5 (Sheet 5.1)
14. Has the same variant/AA change been reported P/LP in ACTC1?	If yes ➔ PS1 (based on the fact, ACTC1 is a fetal skeletal muscle isoform, only 4 AA is different from ACTA1).	Supporting Information 5 (Sheets 5.1 (all actins) and 5.2 (ACTA1 and ACTC1 only))
15. Has a different P/LP AA change been reported at the same residue in any other actin?	If yes ➔ PM5? (based on the fact, actins are highly conserved; highest confidence for ACTC1, then ACTA2/ACTG2, then ACTB/ACTG1).	Supporting Information 5 (Sheet 5.1)
16. Has a different P/LP AA change been reported at the same residue in ACTC1?	If yes ➔ PM5 (based on the fact ACTC1 is a fetal skeletal muscle isoform, only 4 AA is different from ACTA1).	Supporting Information 5 (Sheets 5.1 (all actins) and 5.2 (ACTA1 and ACTC1 only))
17. Have two or more different P/LP changes been reported at the same residue across all actins?	If yes ➔ PM1 (if completely conserved—see above, and no B/LB or gnomAD variants at this position).	Supporting Information 5 (Sheet 5.1)

*Note:* ? = low confidence; requires further discretion.

Abbreviations: AA = amino acid change, P/LP = pathogenic/likely pathogenic.

**Table 3:** Potential *Acta1* mouse models reported in the Mutagenix database.

**Mutagenetix ENU mice**						**VarSome report**		
**Mouse line**	**Base change, position (GRCm38)**	**Amino acid change**	**Zygosity**	**Consequence**	**Classification**	**Variant reported (classification)**	**Position reported (classification)**	**Inheritance**
Z1177	C>A, 123893471	G44V	Heterozygous	Missense, splice acceptor	Probably benign	G44V (P)	—	AD
R9651	A>T, 123892692	Y220Ter	Heterozygous	Nonsense	Probably null	Y220Ter (P)	—	AR
R8336	C>T, 123892571	E261K	Heterozygous	Missense	Possibly damaging	—	E261V (P)	AR
R1901	A>T, 123893161	S147T	Heterozygous	Missense	Probably benign	—	S147F (LP), S147Y (LP)	De novo
R5778	A>G, 123892125	S340P	Heterozygous	Missense	Not run	—	S340W (LP)	Unknown^∗^
R0090	T>C, 123893657	N14S	Heterozygous	Missense	Possibly damaging	—	N14Y (LP)	Unknown^∗^
R2049	G>T, 123892064	T360N	Heterozygous	Missense	Probably benign	—	—	—
R6353	T>C, 123893687	E4G	Heterozygous	Missense	Not run	—	—	—
R6731	G>A, 123893217	T128I	Heterozygous	Missense	Probably damaging	—	—	—
R8070	T>A, 123893621	D26V	Heterozygous	Missense	Possibly damaging	—	—	—
R8826	T>C, 123893239	M121V	Heterozygous	Missense	Probably damaging	—	—	—

^*^Zygosity not reported in the database, likely heterozygous dominant.

## Data Availability

All data used and analyzed as part of this study are available via the Supporting Information.

## Supporting Information

The following Supporting Information are provided alongside this manuscript:

Supporting Information 1 PDF, which includes supporting information (figures and tables).

Supporting Information 2
*ACTA1* variant and phenotype tables. Excel data file, which includes tables of *ACTA1* variants and associated phenotype/s (both simple and detailed). The first “sheet” (labelled “Key”) summarizes the content included in each sheet/table.

Supporting Information 3 Variant analysis and VUS reclassification. Excel data file, which includes tables of variant of uncertain significance (VUS) analysis and reclassification and outputs from various variant analysis tools. The first “sheet” (labelled “Key”) summarizes the content included in each sheet/table.

Supporting Information 4 Mosaics, recessive, and gnomAD. Excel data file, which includes tables of *ACTA1* mosaic cases, recessive cases, and gnomAD variants (including possible recessive variants in gnomAD). The first “sheet” (labelled “Key”) summarizes the content included in each sheet/table.

Supporting Information 5 Pathogenic AA changes in all actins. Excel data file, which includes interactive schematics of a pathogenic amino acid (AA) changes in all actins based on reports from HGMD Pro, LOVD, and ClinVar. The first “sheet” (labelled “Key”) summarizes the content included in each sheet/table and includes additional notes to aid in the use and interpretation of these interactive figures.
