# Supplementary material for: An Update on Reported Variants in the Skeletal Muscle α-Actin (ACTA1) Gene
Source: Hum Mutat. 2024 Oct 28;2024:6496088. doi: 10.1155/2024/6496088 (PMC11918651; doi:10.1155/2024/6496088)
Supplement: Supporting Information 1 — PDF, which includes supporting information (figures and tables). [file 6496088.f1.docx]

## Supplementary Information for article: An update on reported variants in the skeletal muscle α-actin (*ACTA1*) gene

## (Clayton, J et al, 2024)

This file contains supplementary information, figures and tables. In addition, several Supplementary Excel files were prepared for this mutation update. Excel was used due to the volume of data (which would not fit easily into a standard table and therefore not feasible to include as a main data table in the manuscript), and because it allowed the use of ‘sheets’ to collate multiple tables into one document. These files have been included as supplementary files associated with the manuscript, though we have also uploaded these to Microsoft OneDrive and Google Drive to be accessed and/or downloaded – additional folders may be placed in these cloud locations in future if we have capacity to continue to update these tables/resources (if we do so, these will be kept distinct from original manuscript files). Links to these files are provided below. These files will be hosted by Joshua Clayton (first author; contact at [joshua.clayton@perkins.uwa.edu.au](mailto:joshua.clayton@perkins.uwa.edu.au) or [jsjcc26@gmail.com](mailto:jsjcc26@gmail.com)).

Link to Microsoft OneDrive <https://1drv.ms/f/s!AlM6MQoNl8_dhvFFmO5uQN4qFzEfJQ?e=J8IXz3>

Link to Google Drive: <https://drive.google.com/drive/folders/1_p0h_Jx9H9rNFfK-RaocX8WgntjYsWjl?usp=sharing>

***Supplementary data files - titles and descriptions:***

**Supplementary Information 2 – ACTA1 variant and phenotype tables.** Excel file; tables of *ACTA1* variants and associated phenotype/s (both simple and detailed). The first ‘sheet’ (labelled ‘Key’) summarizes the content included in each sheet/table.

**Supplementary Information 3 – Variant analysis, VUS reclassification.** Excel file; tables of variant of Uncertain Significance (VUS) analysis and reclassification, and outputs from various variant analysis tools. The first ‘sheet’ (labelled ‘Key’) summarizes the content included in each sheet/table.

**Supplementary Information 4 – Mosaics, recessive, gnomAD.** Excel file; ables of *ACTA1* mosaic cases, recessive cases, and gnomAD variants (including possible recessive variants in gnomAD). The first ‘sheet’ (labelled ‘Key’) summarizes the content included in each sheet/table.

**Supplementary Information 5 – Pathogenic AA changes in all actins.** Excel file; interactive schematics of pathogenic amino acid (AA) changes in all actins, based on reports from HGMD Pro, LOVD and ClinVar. The first ‘sheet’ (labelled ‘Key’) summarizes the content included in each sheet/table and includes additional notes to aid in use and interpretation of these interactive figures.


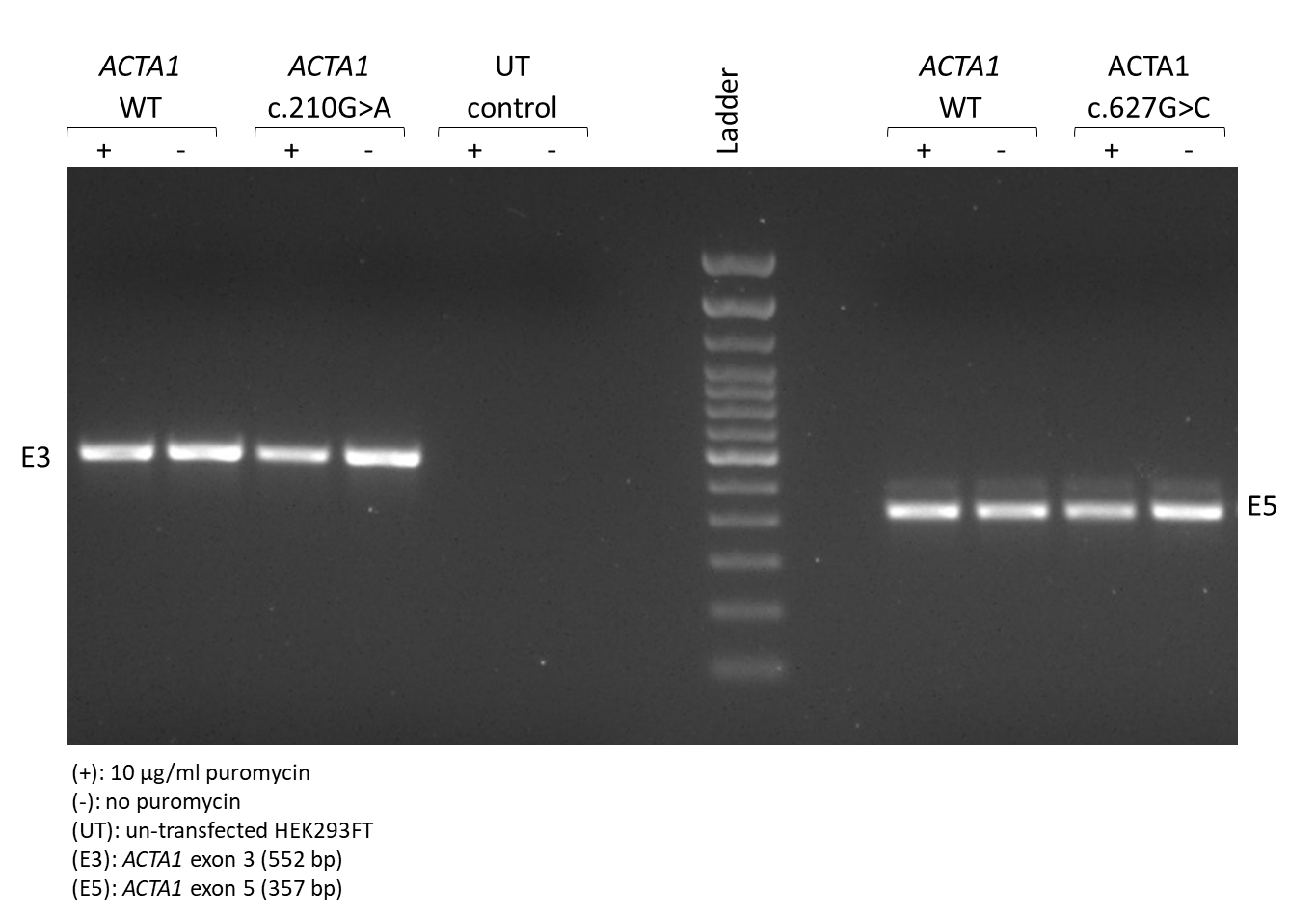


**Supplementary Figure S1. Minigene assays indicate that ACTA1 c.210G>A and c.627G>C variants do not affect splicing.** Agarose gel of RT-PCR products from *ACTA1* minigene assays in HEK293FT cells, conducted in the presence (+) or absence (-) of puromycin [10 µg/mL]. Minigene splicing assays were performed using pCas2.1 constructs containing the exon of interest (exon 3; E3, or exon 5; E5), plus ~150 bp of flanking intronic sequence either side. Expected amplicon sizes were 552 bp for exon 3 (containing c.210G>A) and 357 bp for exon 5 (containing c.627G>C), respectively. No differences in splicing patterns or amplicon size were observed between constructs containing the wildtype *ACTA1* sequence (WT) and those containing the variant, which was also confirmed by Sanger sequencing of RT-PCR products. UT = untransfected control cells (no minigene vector). Puromycin was used to inhibit nonsense-mediated decay. Ladder is TrackIT^TM^ 100 bp DNA ladder (Thermo Fisher).

**Supplementary Information. Identification of a novel c.980T>G (p.Met327Arg) missense variant in *ACTA1*, leading to a genetic diagnosis.**

Patient 1 is a two-year-old male with congenital myopathy who presented with reduced intrauterine fetal movements. He was delivered by Caesarean section and was severely hypotonic. He had axial and peripheral hypotonia, absent deep tendon reflexes and no antigravity movements. He was intubated after birth. His percentiles were below normal and cognitive status appeared normal. Muscle biopsy at 3 months of age showed non-specific myopathic changes and atrophic myofibres. His creatine kinase levels appeared normal (67 U/L; normal upper limit 171 U/L). There is no known consanguinity between the parents, though they originate from close Turkish villages. Whole exome sequencing analysis identified a single candidate - an apparent *de novo* variant in *ACTA1* (c.980T>G, p.(Met327Arg)). Several *in silico* tools predicted this variant to be pathogenic, including CADD (28), SIFT (0.02), MetaSVM (damaging) and Mutation Taster (disease causing). This variant is absent in both confirmed parents and 141,456 individuals in gnomAD. Bidirectional Sanger sequencing of the trio confirmed the *de novo* *ACTA1* c.980T>G variant in the proband (Supp. Fig. S2A).

The p.Met327Arg substitution is a novel change not reported in disease databases (LOVD, Clinvar, Decipher), though two different substitutions at this position were previously reported. Met327Lys (c.980T>A) was identified in a patient with congenital fibre type disproportion (CFTD), and was considered likely pathogenic (Kajino et al., 2014) and subsequently reported in further case with CFTD and dilated cardiomyopathy (Matsumoto et al., 2022). Met327Ile (c.981G>C) was reported in in a patient with congenital myopathy with excess of thin filaments (Clinvar [rs1553255334](https://www.ncbi.nlm.nih.gov/variation/tools/1000genomes/?chr=1&from=229567477&to=229567477&gts=rs1553255334&mk=229567477:229567477|rs1553255334)). Following ACMG-AMP variant interpretation guidelines, we report *ACTA1:*c.980T>A is a class 5 pathogenic variant, as critical threshold requirements were met (Supplementary Table S1).

**References:**

Kajino S, Ishihara K, Goto K, Ishigaki K, Noguchi S, Nonaka I, Osawa M, Nishino I, Hayashi YK. 2014. Congenital fiber type disproportion myopathy caused by LMNA mutations. J Neurol Sci 340:94–98.

Matsumoto A, Tsuda H, Furui S, Kawada-Nagashima M, Anzai T, Seki M, Watanabe K, Muramatsu K, Osaka H, Iwamoto S, Nishino I, Yamagata T. 2022. A case of congenital fiber-type disproportion syndrome presenting dilated cardiomyopathy with ACTA1 mutation. Mol Genet Genomic Med 10:1–6.

**Supplementary Table S1. ACMG-AMP Guidelines for classification of *ACTA1:*c.980T>A as class 5; pathogenic given the following requirements were met: Strong x1 AND moderate x2 AND supporting x2.**

| Classification | Evidence of pathogenicity | Explanation |
| --- | --- | --- |
| PS2 | Strong | *De novo* variant in a patient that is absent in confirmed maternal and paternal samples (strong if paternity/maternity confirmed) with no family history |
| PM2 | Moderate | Variant is absent from controls in gnomAD |
| PM5 | Moderate | Novel missense variant at a position where a different missense variant has been determined as pathogenic |
| PP2 | Supporting | Missense variant in gene where missense variants are a common cause of disease, and low frequency of benign missense variants observed |
| PP3 | Supporting | Multiple computational tools predict a deleterious effect. |


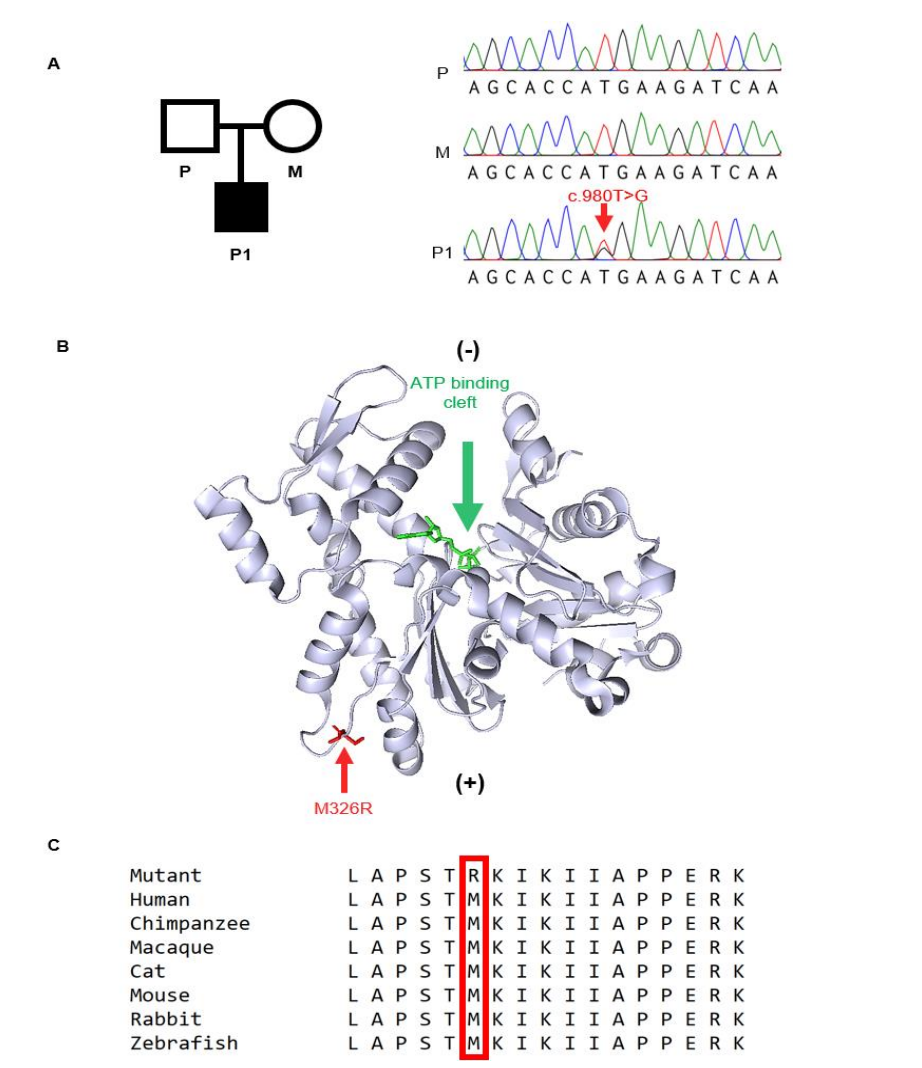


**Supplementary Figure S2**. Summary of the *ACTA1* c.980T>G (p.Met327Arg) variant identified in Patient 1 (P1). (A) Pedigree (left) of P1 and unaffected Paternal (P) and Maternal (M) parents. Sanger sequencing chromatogram (right) confirms the de novo c.980T>G variant is absent in both parents. (B) Structural modelling of a single α-actin monomer (G-actin) in rabbit (PDB 4B1X). Location of the substitution (M326R in rabbit) is shown in relation to the barbed (+) and pointed (-) ends of G-actin. (C) Conservation of methionine 327 (M) in various species.

**Supplementary Table S2. Conditions caused by P/LP variants in *ACTA1* (reports with verified phenotypes from HGMD/LOVD only).**

|  | **Condition** | **# variants causing** | **% of total reports**  **(n = 304 variants)** |
| --- | --- | --- | --- |
|  | Nemaline myopathy | 225 | 74.0% |
|  | Congenital fibre type disproportion | 22 | 7.2% |
|  | Congenital myopathy | 20 | 6.6% |
|  | Myopathy | 18 | 5.9% |
|  | Intranuclear rod myopathy | 13 | 4.3% |
|  | Dilated cardiomyopathy | 11 | 3.6% |
|  | Arthrogryposis multiplex congenita | 9 | 2.6% |
|  | Actin myopathy | 7 | 2.3% |
|  | Fetal akinesia deformation sequence | 6 | 2.0% |
|  | Hypertrophic cardiomyopathy | 5 | 1.6% |
|  | Limb-girdle muscular dystrophy | 4 | 1.3% |
|  | Muscular dystrophy | 3 | 1.0% |
|  | Neuromuscular disease | 3 | 1.0% |
|  | Muscle weakness | 3 | 1.0% |
|  | Distal myopathy | 2 | 0.7% |
|  | Zebra body myopathy | 2 | 0.7% |
|  | Myofibrillar myopathy | 2 | 0.7% |
|  | Cap myopathy | 2 | 0.7% |
|  | Congenital muscular dystrophy | 2 | 0.7% |
|  | Core myopathy | 2 | 0.7% |
|  | Cytoplasmic body myopathy | 2 | 0.7% |
|  | Cardiomyopathy | 1 | 0.3% |
|  | Inclusion-body myositis | 1 | 0.3% |
|  | Hydrops fetalis | 1 | 0.3% |
|  | Facioscapuloperoneal myopathy | 1 | 0.3% |
|  | Rigid spine muscular dystrophy | 1 | 0.3% |
|  | Progressive scapuloperoneal myopathy | 1 | 0.3% |
|  | Scapulohumeralperoneal myopathy | 1 | 0.3% |
|  | Developmental disorder | 1 | 0.3% |
|  | Fetal abnormalities | 1 | 0.3% |

*Yellow: skeletal myopathy, Red: cardiomyopathy, Blue: fetal abnormalities, Green: muscular dystrophy.

Note 1: broad disease categories such as “Congenital myopathy”, “Muscular dystrophy” and “Cardiomyopathy” refer to cases where more a more precise phenotype has not been ascribed.

Note 2: Numbers in table are calculated from number of variant-disease associations i.e. does not reflect ‘unique’ cases (as there are several variants with multiple disease associations).

Note 3: Numbers are calculated from variants with phenotype report in LOVD and/or HGMD. ClinVar reports typically do not report specific phenotype (most simply report “Actin aggregate myopathy”) and many reports are spurious (i.e. affected status unconfirmed). There are a few valid cases in ClinVar but for consistency decided easiest to just exclude these.

**Supplementary Table S3. Variants used to construct phenotypic overlap diagram (Main Figure 6).** Table output from <https://bioinformatics.psb.ugent.be/webtools/Venn/>.

| **Phenotype** | **total** | **Variant** |
| --- | --- | --- |
| Fetal abnormality/ Skeletal myopathy | 6 | c.44G>A c.226G>C c.215C>G c.739G>A c.449C>T c.220G>A |
| Cardiomyopathy/  Skeletal myopathy | 9 | c.715G>A c.766C>G c.172G>A c.1007A>C c.980T>A c.1074G>T c.880G>T c.143G>C c.1012A>G |
| Muscular dystrophy/  Skeletal myopathy | 5 | c.889G>A c.437C>T c.923A>G c.1057A>G c.493G>T |
| Fetal abnormality | 10 | c.440C>A c.116G>A c.210G>T c.352C>A c.767G>T c.442G>C c.468C>A c.50G>A c.675C>A c.110T>G |
| Cardiomyopathy | 7 | c.767G>A c.848G>A c.1029C>G c.472G>A c.44G>T c.929G>A c.607G>A |
| Muscular dystrophy | 4 | c.723C>A c.440C>T c.460G>C c.341C>T |
| Skeletal myopathy | 262 | c.541G>A c.79G>A c.984G>C c.253G>A c.881A>T c.541G>C c.1043T>A c.360G>T c.1006G>A c.553C>G c.1013A>T c.1049C>T c.128A>G c.557A>G c.355G>C c.407T>C c.739G>C c.925C>T c.760A>C c.1061T>A c.133G>T c.667C>G c.553C>T c.275_277del c.902T>A c.595G>A c.1125A>C c.413T>C c.764A>G c.772C>G c.975C>A c.419C>A c.649A>T c.437_442dupCCTCCG c.443G>T c.449C>G c.142G>C c.551G>A c.983_985del c.794A>T c.627G>C c.808G>C c.145A>G c.197T>A c.616G>A c.677A>G c.818C>G c.742C>A c.148G>T c.868G>A c.338A>G c.758G>A c.442G>A c.863A>G c.570C>A c.1114G>T c.593G>A c.1009C>G c.616+2T>C c.695C>T c.414C>G c.1106C>A c.854T>G c.400A>G c.630C>G c.1130T>G c.800C>G c.217A>G c.808G>A c.773G>A c.770T>G c.749T>C c.556G>T c.296C>G c.149G>T c.422T>C c.802T>C c.687G>C c.911del c.113G>C c.743A>G c.142G>A c.209A>G c.235A>G c.364A>C c.346G>T c.130-5T>A c.435C>G c.417G>C c.119C>T c.194G>A c.466G>A c.83C>A c.760A>T c.478G>A c.593G>T c.682G>C c.224A>G c.466G>C c.393del c.556G>C c.668T>C c.169G>C c.536G>T c.591G>T c.586A>C c.16G>A c.418G>C c.402G>T c.143G>A c.646G>C c.1111A>C c.436del c.487C>G c.109G>C c.539T>C c.782A>T c.37G>A c.1133A>G c.871A>T c.1123A>G c.1019C>G c.1132T>C c.89G>A c.1000C>T c.1061T>C c.621G>C c.676G>C c.505G>A c.109G>T c.327G>C c.223C>A c.282C>A c.131G>T c.347C>T c.49G>A c.682G>T c.599A>G c.542A>G c.1106C>T c.365C>G c.515C>A c.350A>C c.614C>A c.737A>C c.197T>G c.868G>C c.60A>C c.874A>G c.346G>C c.1111A>T c.727G>A c.515C>G c.1123A>C c.809-2A>T c.203C>A c.990+1G>T c.343A>G c.210G>A c.7G>T c.611C>T c.283G>A c.541del c.317T>C c.239A>G c.529A>G c.455-1G>A c.142G>T c.1076T>C c.920T>G c.1031delG c.350A>G c.587C>T c.203C>T c.547G>A c.121C>T c.572T>C c.49G>C c.685A>G c.217A>T c.1001C>T c.455G>C c.124C>T c.796C>A c.686T>C c.617-5C>A c.175G>A c.814G>C c.448A>G c.1013A>C c.971C>G c.346G>A c.757G>C c.715G>T c.460G>T c.493G>C c.1128C>A c.807_808delCGinsAA c.229A>C c.137T>C c.834G>T c.1001C>G c.808G>T c.687G>A c.591G>C c.821C>A c.446G>A c.222_223delGCinsTT c.1099G>A c.224A>T c.888T>G c.227G>A c.773G>T c.425T>C c.449C>A c.1004C>T c.1134G>T c.1127G>C c.478G>T c.149G>A c.824G>C c.809G>A c.1130T>A c.598T>C c.846C>G c.487C>T c.1092delC c.687G>T c.616+1G>A c.821C>T c.493G>A c.812T>G c.738C>A c.1006G>C c.1008G>C c.15C>G c.1120C>A c.443G>A c.553C>A c.112G>A c.230T>G c.148G>A c.811A>G c.287T>C c.243G>A c.592C>A c.579G>T c.110T>C c.554G>T c.592C>T c.1120C>T c.809-1G>T c.353G>A c.854T>A c.122G>A c.841T>C c.956T>C c.(?_-12)_(*254_?)del c.282C>G c.402G>A c.1075A>C c.620A>G |

**Supplementary Table S4. New phenotypes associated with *ACTA1* variants.**

| **Condition/phenotype** | **Notable clinical features** | ***ACTA1* variant/s** | **Effect of mutation** | **# patients, inheritance** | **Additional notes** | **Reference** |
| --- | --- | --- | --- | --- | --- | --- |
| ACTA1-myopathy with predominant finger flexor weakness and rimmed vacuoles | Proband (39yo/F) had delayed motor milestones, weakness. Biopsy: rimmed vacuoles, type 1 fibre atrophy.  Father previously mis-diagnosed (at 54) with sporadic inclusion-body myositis (sIBM)*. Subsquent biopsy showed nemaline rods and did not identify tubulofilamentous inclusions. | c.149G>A (p.Gly50Asp) | Dominant | n = 2 | Two patients with rimmed vacuoles accompanying ACTA1-myopathy. | (Liewluck et al., 2019) |
| Cap myopathy | 5wk: non-specific myopathic features, small fibres.  4yo: Biopsy showed sarcolemally-located caps immunopositive for alpha-actinin and actin; caps present in almost every myofibre (ie developed later in life). | c.145A>G  (p.Met49Val)  [p.Met47Val in paper] | Dominant | n = 1 (de novo) | Variant affects DNA binding loop of the protein. | (Hung et al., 2010) |
| Congenital muscular dystrophy with rigid spine | Generalised weakness in first few years of life. Biopsy (17yo): fibre size variation, numerous internalised nuclei, fibre splitting, occasional degenerating and regenerating fibres, smeared and accumulated Z-band material, occasional small nemaline rods. | c.460G>C (p.Val154Leu) | Recessive | n = 2 | Cardiac actin present in 20% of myofibres. | (O’Grady et al., 2015) |
| Cytoplasmic body myopathy | P1, at birth: hypotonia, severe weakness and respiratory insufficiency. No definitive evidence of nemaline rods.  P1/P2, after birth: marked hypotonia, generalised muscle weakness, respiratory insufficiency. 2mo: fibre size variation, polygonal and rounded atrophy, occasional internal nuclei. | c.282C>A (p.Asn94Lys) | Dominant | n = 3 | Two unrelated families, including two fraternal twins. Mutation previously reported in case of severe NEM. | (Donkervoort et al., 2017) |
| Cytoplasmic body myopathy | 54yr ambulatory patient, absent nemaline rods. Presented at 14yo with general muscle atrophy and weakness. Abundant cytoplasmic bodies in quadriceps. | c.275_277delTCT (p.Phe92del) | Dominant | n = 1 (parents not available) | Variant in domain that forms part of actin-myosin interface with Tyr93/Arg97 at centre. | (Schuelke et al., 2018) |
| Distal myopathy (childhood-to-juvenile onset) | Proband (68yo male): delayed motor milestones and progressive limb weakness since early childhood. Biopsy (triceps) showed numerous highly atrophic fibers, increased internal nuclei, scattered necrotic and regenerating fibres, some fibre splitting and mild inflammatory exudate. Only 2 fibres out of 1000 contained nemaline rods. Affected daughter showed abundant nemaline bodies, core-like structures and type 1 fibre predominance. | c.757G>C (p.Gly253Arg) | Dominant | n = 3 (patient, two children) | Phenotype overlaps with reports in (Zukosky et al., 2015). | (Liewluck et al., 2017) |
| Dominant cardiomyopathy without skeletal myopathy | Family history of cardiac dysfunction. Left ventricle (LV) dysfunction. Proband and two other affected family members negative on diagnostic testing for cardiomyopathy-associated genes. | c.767G>A (p.Arg256His) | Dominant | n = 7 (3 generations) | Unique case/s of dominant cardiomyopathy without functional skeletal myopathy associated with *ACTA1* variant. | (Reza et al., 2018) |
| Inclusion-body myopathy* | French cohort of *GNE*-mutation negative inclusion body myopathy patients. Patient showed distal myopathy of lower limbs with very slow evolution. Rimmed vacuoles present on muscle biopsy. | c.437C>T (p.Ala146Val) | Dominant | n = 1 | *ACTA1* variant here was described only as “possible diagnosis”. | (Cerino et al., 2017) |
| Myofibrillar myopathy | Childhood-onset, slowly progressive limb girdle muscle weakness, typical myofibrillar muscle pathology. Diagnosed at 23 yo with MFM based on muscle biopsy showing retention of granular cytoplasmic labelling for desmin and myotilin and presence of rimmed vacuoles. EM: large areas of Z-line material, dense osmiophilic granular areas, filamentous material. | c.749T>C (p.Ile250Thr) | Recessive | n = 2 | New autosomal recessive form of myofibrillar myopathy. | (Guglieri et al., 2014) |
| Myofibrillar myopathy | Child (26mo) with hypotonia, significant motor milestone delay, severe muscle weakness. Biopsy shoed abnormal variation in fibre size, fibre splitting, increased internal nuclei, vacuolar change, focal areas of myofibrillar disorganisation or hyaline structures. Many structurally abnormal fibres displayed ectopic or abnormal expression of desmin. | c.437_442dup (p.Ala146_Ser147dup) | Dominant | n = 1 | Contrary to most MFM patients (non-ACTA1), patient here presented in childhood. | (Selcen, 2015) |
| Scapuloperoneal myopathy (scapulo-humeral-peroneal-distal) | Largest actinopathy pedigree to date. Different to other actinopathies clinically and morphologically; scapulo-humeral-peroneal-distal distribution with striking upper extremity predilection in some individuals, progressive but variable course of diseases, sparing of respiratory muscles until very late in disease. No nemaline rods, even in advanced stages of disease. | c.591C>A (p.Glu197Asp) | Dominant | n = 14 examined (n = 33 total individuals) | Large multi-generational family with extensive clinical examination. | (Zukosky et al., 2015) |
| Scapuloperoneal myopathy | Same clinical phenotype and mutation as Zukosky et al. (2015). Unlike family described by Zukosky et al, cases showed abundance of nemaline rods. | c.591C>A (p.Glu197Asp) | Dominant | n = 2 (mother, son) | Same clinical features/mutation as (Zukosky et al., 2015) but rods present. | (Hernandez-Lain et al., 2019) |
| Zebra body myopathy | Proband hypotonic at birth, weak fetal movements. Biopsy: wide variation in fibre size, type 1 fibre predominance, myofibrillar disorganisation, nemaline rods, myelin-like whorls in vacuolated fibres, numerous Zebra bodies. Second biopsy showed only a few Zebra bodies. | c.1043T>A (p.Leu348Gln) | Dominant | n = 1  (likely de novo; parents unaffected) | Historical case from 1975 reclassified as zebra body myopathy. | (Sewry et al., 2015) |

*Note: This case of IBM was reported in HGMD, but has since been updated to remove this citation (Cerino et al., 2017). Another case of sporadic inclusion-body myositis was also mentioned in (Liewluck et al., 2019), which is acknowledged by the authors to be a mis-diagnosis based mainly on the pattern of weakness observed (thumb or finger flexors were weaker than finger flexors and knee extensors and hip flexors were equally weak). This, along with the rimmed vacuoles on biopsy led to mis-diagnosis, despite the young age of disease onset arguing against sIBM. The identification of the *ACTA1* variant in their similarly-affected offspring led to clarification as *ACTA1*-myopathy with prominent finger flexor weakness and rimmed vacuoles.

**Supplementary Table S5. New co-pathologies associated with *ACTA1* variants.**

| **Condition/phenotype** | **Notable clinical features** | **ACTA1 variant/s** | **Effect of mutation** | **# patients, inheritance** | **Additional notes** | **Reference** |
| --- | --- | --- | --- | --- | --- | --- |
| Congenital fibre-type disproportion (CFTD) with dilated cardiomyopathy | Low muscle tone and delayed motor development in early years. At 19yo, long face, high palate, mild muscle weakness and atrophy in all extremities and trunk with decreased deep tendon reflexes. MRI findings indicated dilated cardiomyopathy. Biopsy (left bices brachii): predominant type 1 fibre atrophy, without any other significant pathological findings (nemaline rods etc.). | c.143G>C (p.Gly48Ala) | Dominant | n = 1  (de novo) | Novel *ACTA1* mutation causing CFTD accompanied by dilated cardiomyopathy. | (Tadokoro et al., 2018) |
| Congenital fibre type disproportion (CFTD) with dilated cardiomyopathy | Proband (10yo/M) with CFTD and dilated cardiomyopathy. Biopsy (left quadriceps): bimodal distribution of fibre size. Did not detect rimmed vacuoles, nemaline bodies or ragged-red fibres. | c.980T>A (p.Met327Lys) | Dominant | n = 1  (de novo) | Rare finding of cardiomyopathy accompanying CFTD. | (Matsumoto et al., 2022) |
| Dilated cardiomyopathy with childhood-onset nemaline myopathy | Somewhat atypical presentation of childhood-onset nemaline myopathy. Biopsy (left triceps brachii): mild variation of muscle fibre size, multiple cytoplasmic nemaline rods in some fibres, no myofibrillar abnormalities or cores. | c.1074G>T (p.Trp358Cys) | Dominant | n = 1 | First reported case of dilated cardiomyopathy associated with childhood-onset NEM. | (Gatayama et al., 2013) |
| Nemaline myopathy with mitochondrial complex I deficiency | 1mo: Myopathic facies, micrognathia, high arched palate. 2yo: reduced muscle strength, breathing difficulties. Type I myofibre predominance, rod-like inclusions. Normal levels of NDUFB8 and COX1. | c.760A>C (p.Asn254His) | Dominant | n = 1  (de novo) | May not be new – paper notes Lamont et al. 2004, which includes 3 cases of NM with complex I deficiency | (Pula et al., 2020) |
| Nemaline myopathy with stiffness and hypertonia | 6wk: first presentation. 10wk: Hypertonia (abdominal and proximal limb muscles), episodic muscle stiffness, contractures in elbows and knees. Died at 9mo. | c.984G>C (p.Lys328Asn) | Dominant | n = 1  (de novo) | Mutation increases activated states of the thin filament. | (Jain et al., 2012) |
| Nemaline myopathy with myofibrillar dysgenesis and abnormal ossification | 2yo/M with marked hypotonia, ventilator-dependent since birth. Biopsy: cytoplasmic accumulation of thin filament aggregates, marked myofibrillar dysgenesis, intranuclear rods and dispersed tiny nemaline bodies. Craniofacial sclerosis and longitudinal striations in iliac bone. | c.430C>T (p.Leu144Phe) | Dominant | n = 1 (de novo) | Focus on extramucular manifestations i.e. abnormal ossification. | (Arai et al., 2009) |
| Late-presenting nemaline myopathy with unusual dark cores | Mild myopathic face, atrophy of sternocleidomastoideus and temporalis muscles, neck flexor weakness, diffuse muscle weakness. Muscle biopsy showed atypical dark characterised by dark material with a peripheral halo upon staining with Gomori trichrome. | c.148G>A (p.Gly50Ser) | Dominant | n = 6 (3 generations) | Peculiar morphological elements at muscle biopsy. | (Garibaldi et al., 2021) |
| Severe congenital nemaline myopathy with primary pulmonary lyphangiectasia | Affected newborn with no spontaneous movement, fractures at birth, respiratory insufficiency. Developed bilateral chylothorax at two weeks and died at 6 weeks. Biopsy/EM = diagnosis of NEM. Histological examination of both lungs revealed primary pulmonary lymphangiectasia. | c.1127G>C (p.Cys376Ser) | Dominant | n = 1 | Unusual clinical presentation of severe congenital NEM (bilateral chylothrax). | (Waisayarat et al., 2015) |

**Supplementary Table S6. Other interesting phenotypes associated with *ACTA1* variants.**

| **Condition/phenotype** | **Notable clinical features** | **ACTA1 variant/s** | **Effect of mutation** | **# patients, inheritance** | **Additional notes** | **Reference** |
| --- | --- | --- | --- | --- | --- | --- |
| Congenital myopathy with variably-penetrant cardiomyopathy | All affected individuals (n=4) hypotonic at birth, delayed motor milestones and progressive weakness in grip and proximal muscle strength over time. Prominent finger flexor weakness, CFTD, and cardiac structural abnormalities along the spectrum of left ventricular dilation and systolic dysfunction with associated conduction defects and arrhythmias. | c.81C>A (p.Asp27Glu) | Dominant | n = 4 (3 generations) | Two infrequently-observed clinical features in *ACTA1*-myopathy: deep finger flexor weakness, and cardiac structural and conduction disease. | (Mulvany-Robbins et al., 2023) |

**Supplementary table references:**

Arai A, Mitsuhashi S, Saito Y, Komaki H, Sakuma H, Nakagawa E, Sugai K, Sasaki M, Robertson SP, Nishimura G, Yamamoto T, Nonaka I, et al. 2009. Nemaline (actin) myopathy with myofibrillar dysgenesis and abnormal ossification. Neuromuscul Disord 19:485–488.

Cerino M, Gorokhova S, Laforet P, Yaou R Ben, Salort-Campana E, Pouget J, Attarian S, Eymard B, Deleuze JF, Boland A, Behin A, Stojkovic T, et al. 2017. Genetic Characterization of a French Cohort of GNE-mutation negative inclusion body myopathy patients with exome sequencing. Muscle and Nerve 56:993–997.

Donkervoort S, Chan SHS, Hayes LH, Bradley N, Nguyen D, Leach ME, Mohassel P, Hu Y, Thangarajh M, Bharucha-Goebel D, Kan A, Ho RSL, et al. 2017. Cytoplasmic body pathology in severe ACTA1-related myopathy in the absence of typical nemaline rods. Neuromuscul Disord 27:531–536.

Garibaldi M, Fattori F, Pennisi EM, Merlonghi G, Fionda L, Vanoli F, Leonardi L, Bucci E, Morino S, Micaloni A, Tartaglione T, Uijterwijk B, et al. 2021. Novel ACTA1 mutation causes late-presenting nemaline myopathy with unusual dark cores. Neuromuscul Disord 31:139–148.

Gatayama R, Ueno K, Nakamura H, Yanagi S, Ueda H, Yamagishi H, Yasui S. 2013. Nemaline Myopathy With Dilated Cardiomyopathy in Childhood. Pediatrics 131:e1986–e1990.

Guglieri M, Sambuughin N, Sarkozy A, Barresi R, Lochmüller H, Bushby K, Goldfarb L, Straub V. 2014. A.P.6 Autosomal recessive myofibrillar myopathy caused by ACTA1 mutations. Neuromuscul Disord 24:832.

Hernandez-Lain A, Cantero D, Camacho-Salas A, Toldos O, Esteban I, Pascual I, Dominguez-Gonzalez C. 2019. Autosomal dominant distal myopathy with nemaline rods due to p.Glu197Asp mutation in ACTA1. Neuromuscul Disord 29:247–250.

Hung RM, Yoon G, Hawkins CE, Halliday W, Biggar D, Vajsar J. 2010. Cap myopathy caused by a mutation of the skeletal alpha-actin gene ACTA1. Neuromuscul Disord 20:238–240.

Jain RK, Jayawant S, Squier W, Muntoni F, Sewry CA, Manzur A, Quinlivan R, Lillis S, Jungbluth H, Sparrow JC, Ravenscroft G, Nowak KJ, et al. 2012. Nemaline myopathy with stiffness and hypertonia associated with an ACTA1 mutation. Neurology 78:1100–1103.

Labasse C, Brochier G, Taratuto A-L, Cadot B, Rendu J, Monges S, Biancalana V, Quijano-Roy S, Bui MT, Chanut A, Madelaine A, Lacène E, et al. 2022. Severe ACTA1-related nemaline myopathy: intranuclear rods, cytoplasmic bodies, and enlarged perinuclear space as characteristic pathological features on muscle biopsies. Acta Neuropathol Commun 10:101.

Liewluck T, Niu Z, Moore SA, Alsharabati M, Milone M. 2019. ACTA1-myopathy with prominent finger flexor weakness and rimmed vacuoles. Neuromuscul Disord 29:388–391.

Liewluck T, Sorenson EJ, Walkiewicz MA, Rumilla KM, Milone M. 2017. Autosomal dominant distal myopathy due to a novel ACTA1 mutation. Neuromuscul Disord 27:742–746.

Matsumoto A, Tsuda H, Furui S, Kawada-Nagashima M, Anzai T, Seki M, Watanabe K, Muramatsu K, Osaka H, Iwamoto S, Nishino I, Yamagata T. 2022. A case of congenital fiber-type disproportion syndrome presenting dilated cardiomyopathy with ACTA1 mutation. Mol Genet Genomic Med 10:1–6.

Mulvany-Robbins B, Putko B, Schmitt L, Oudit G, Phan C, Beecher G. 2023. Novel p.Asp27Glu ACTA1 variant features congenital myopathy with finger flexor weakness, cardiomyopathy, and cardiac conduction defects. Neuromuscul Disord 33:546–550.

O’Grady GL, Best HA, Oates EC, Kaur S, Charlton A, Brammah S, Punetha J, Kesari A, North KN, Ilkovski B, Hoffman EP, Clarke NF. 2015. Recessive ACTA1 variant causes congenital muscular dystrophy with rigid spine. Eur J Hum Genet 23:883–886.

Pula S, Urankar K, Norman A, Pierre G, Langton-Hewer S, Selby V, Mason F, Vijayakumar K, McFarland R, Taylor RW, Majumdar A. 2020. A novel de novo ACTA1 variant in a patient with nemaline myopathy and mitochondrial Complex I deficiency. Neuromuscul Disord 30:159–164.

Reza N, Garg A, Merrill SL, Chowns JL, Rao S, Owens AT. 2018. ACTA1 Novel Likely Pathogenic Variant in a Family With Dilated Cardiomyopathy. Circ Genomic Precis Med 11:e002243.

Ross JA, Levy Y, Ripolone M, Kolb JS, Turmaine M, Holt M, Lindqvist J, Claeys KG, Weis J, Monforte M, Tasca G, Moggio M, et al. 2019. Impairments in contractility and cytoskeletal organisation cause nuclear defects in nemaline myopathy. Acta Neuropathol 138:477–495.

Schuelke M, Schwarz M, Stenzel W, Goebel HH. 2018. Cytoplasmic body myopathy revisited. Neuromuscul Disord 28:969–971.

Selcen D. 2015. Severe congenital actin related myopathy with myofibrillar myopathy features. Neuromuscul Disord 25:488–492.

Sewry CA, Holton JL, Dick DJ, Muntoni F, Hanna MG. 2015. Zebra body myopathy is caused by a mutation in the skeletal muscle actin gene (ACTA1). Neuromuscul Disord 25:388–391.

Tadokoro K, Ohta Y, Sasaki R, Takahashi Y, Sato K, Shang J, Takemoto M, Hishikawa N, Yamashita T, Nakamura K, Nishino I, Abe K. 2018. Congenital myopathy with fiber-type disproportion accompanied by dilated cardiomyopathy in a patient with a novel p.G48A ACTA1 mutation. J Neurol Sci 393:142–144.

Waisayarat J, Suriyonplengsaeng C, Khongkhatithum C, Rochanawutanon M. 2015. Severe congenital nemaline myopathy with primary pulmonary lymphangiectasia: Unusual clinical presentation and review of the literature. Diagn Pathol 10:4–9.

Zukosky K, Meilleur K, Traynor BJ, Dastgir J, Medne L, Devoto M, Collins J, Rooney J, Zou Y, Yang ML, Gibbs JR, Meier M, et al. 2015. Association of a Novel ACTA1 Mutation With a Dominant Progressive Scapuloperoneal Myopathy in an Extended Family. JAMA Neurol 72:689.
